# Blocking Y-Box Binding Protein-1 through Simultaneous Targeting of PI3K and MAPK in Triple Negative Breast Cancers

**DOI:** 10.3390/cancers12102795

**Published:** 2020-09-29

**Authors:** Aadhya Tiwari, Mari Iida, Corinna Kosnopfel, Mahyar Abbariki, Apostolos Menegakis, Birgit Fehrenbacher, Julia Maier, Martin Schaller, Sara Y. Brucker, Deric L. Wheeler, Paul M. Harari, Ulrich Rothbauer, Birgit Schittek, Daniel Zips, Mahmoud Toulany

**Affiliations:** 1 Division of Radiobiology and Molecular Environmental Research, Department of Radiation Oncology, University of Tuebingen, 72076 Tuebingen, Germany; Atiwari3@mdanderson.org (A.T.); daniel.zips@med.uni-tuebingen.de (D.Z.); 2 Department of Radiation Oncology, University of Tuebingen, 72076 Tuebingen, Germany; a.menegakis@nki.nl; 3 German Cancer Consortium (DKTK), Partner Site Tuebingen and German Cancer Research Center (DKFZ), Im Neuenheimer Feld 280, 69120 Heidelberg, Germany; 4 Department of Human Oncology, University of Wisconsin, Madison, WI 53705, USA; iida@humonc.wisc.edu (M.I.); abbariki@wisc.edu (M.A.); dlwheeler@wisc.edu (D.L.W.); harari@humonc.wisc.edu (P.M.H.); 5 Department of Dermatology, University of Tuebingen, 72076 Tuebingen, Germany; Kosnopfel_C@ukw.de (C.K.); Birgit.Fehrenbacher@med.uni-tuebingen.de (B.F.); Martin.Schaller@med.uni-tuebingen.de (M.S.); birgit.schittek@uni-tuebingen.de (B.S.); 6 Natural and Medical Sciences Institute, University of Tuebingen, 72770 Reutlingen, Germany; julia.maier11@googlemail.com (J.M.); ulrich.rothbauer@uni-tuebingen.de (U.R.); 7 Pharmaceutical Biotechnology, University of Tuebingen, 72076 Tuebingen, Germany; 8 Department of Women’s Health, University of Tuebingen, 72076 Tuebingen, Germany; sara.brucker@med.uni-tuebingen.de

**Keywords:** triple negative breast cancer cells, KRAS, PTEN, PIK3CA, YB-1, MAPK/ERK, PI3K/Akt

## Abstract

**Simple Summary:**

Triple-negative breast cancer (TNBC) is associated with the high rates of relapse and metastasis and poor survival. YB-1 is overexpressed in TNBC tumor tissues. In the present study, we demonstrated that S102 phosphorylation of YB-1 in TNBC cell lines depend on the mutation status of the components of the MAPK/ERK and PI3K/Akt pathways. Simultaneous targeting of MEK and PI3K was found to be the most effective approach to block YB-1 phosphorylation and to inhibit YB-1 dependent cell proliferation. *YBX1* knockout was sufficient to block TNBC tumor growth.

**Abstract:**

The multifunctional protein Y-box binding protein-1 (YB-1) regulates all the so far described cancer hallmarks including cell proliferation and survival. The MAPK/ERK and PI3K/Akt pathways are also the major pathways involved in cell growth, proliferation, and survival, and are the frequently hyperactivated pathways in human cancers. A gain of function mutation in *KRAS* mainly leads to the constitutive activation of the MAPK pathway, while the activation of the PI3K/Akt pathway occurs either through the loss of PTEN or a gain of function mutation of the catalytic subunit alpha of PI3K (*PIK3CA*). In this study, we investigated the underlying signaling pathway involved in YB-1 phosphorylation at serine 102 (S102) in *KRAS(G13D)*-mutated triple-negative breast cancer (TNBC) MDA-MB-231 cells versus *PIK3CA(H1047R)*/*PTEN(E307K)* mutated TNBC MDA-MB-453 cells. Our data demonstrate that S102 phosphorylation of YB-1 in *KRAS*-mutated cells is mainly dependent on the MAPK/ERK pathway, while in *PIK3CA*/*PTEN*-mutated cells, YB-1 S102 phosphorylation is entirely dependent on the PI3K/Akt pathway. Independent of the individual dominant pathway regulating YB-1 phosphorylation, dual targeting of MEK and PI3K efficiently inhibited YB-1 phosphorylation and blocked cell proliferation. This represents functional crosstalk between the two pathways. Our data obtained from the experiments, applying pharmacological inhibitors and genetic approaches, shows that YB-1 is a key player in cell proliferation, clonogenic activity, and tumor growth of TNBC cells through the MAPK and PI3K pathways. Therefore, dual inhibition of these two pathways or single targeting of YB-1 may be an effective strategy to treat TNBC.

## 1. Introduction

Y-box binding protein-1 (YB-1), encoded by the *YBX1* gene, is a multifunctional protein that participates in DNA repair, gene transcription, mRNA splicing, and translation [1]. YB-1 is one of the rare proteins that regulates the cellular signaling pathways underlying nearly every cancer hallmark [2,3]. As both a cytoplasmic and nuclear protein, YB-1 is highly expressed in different cancer types, such as breast, lung, colorectal, melanocytic, prostate, ovary, and bone cancer [2,4,5,6,7]. In breast carcinomas, expression of cytoplasmic YB-1 has been shown to be associated with tumor aggressiveness, and nuclear localization was shown to be a predictive marker of recurrence after chemo- and radiotherapy [8]. Nuclear localization was also associated with increased tumor grading and tumor stage in breast cancer [9]. For most of its functions, YB-1 must be phosphorylated at serine residue 102 (S102), and the level of phosphorylation is directly correlated with poor clinical outcomes, e.g., in lymphoma patients [10]. Previous reports have demonstrated that signaling pathways downstream of ERK regulate YB-1 S102 phosphorylation [11,12,13]. It has been reported that p90 ribosomal S6 kinase (RSK), which acts downstream of ERK activity, is the major kinase regulating YB-1 phosphorylation at S102 [14,15]. Recently, we reported that phosphorylated YB-1 does not translocate to the nucleus [16]. Instead, phosphorylation of nuclear YB-1 after various cellular stress, e.g., ligand stimulation, irradiation, and expression of *KRAS(G12V)*, is regulated by nuclear translocation of phospho-RSK [16]. Therefore, targeting RSK could be an efficient approach for eliminating tumor-initiating cells via YB-1 inactivation in triple-negative breast cancer (TNBC) [15] lacking the expression of estrogen, progesterone, and HER2 receptors.

KRAS is a small GTPase that is downstream of several membrane-bound receptors, such as the receptor tyrosine kinases that transduce extracellular signals into cells. Approximately 85% of mutations in RAS isoforms (*KRAS*, *HRAS* and *NRAS*) occur in *KRAS* and are associated with unfavorable prognoses [17]. Different point mutations in the *KRAS* gene differentially affect cellular functions. For instance, the *KRAS(G12V)* mutation stimulates metastasis in colorectal cancer models in a manner much stronger than the *KRAS(G13D)* mutation [18]. In a previous study, we demonstrated for the first time that exposure to a clinically relevant dose of ionizing radiation (IR) induces YB-1 phosphorylation at S102 in *KRAS* wild-type breast cancer cells, detected up to 30 min after irradiation [19]. IR-induced YB-1 phosphorylation was shown to be markedly dependent on activation of epidermal growth factor receptor (EGFR) and the MAPK/ERK and PI3K/Akt pathways [19], the pathways that are known to be upregulated in *KRAS* mutated cells [20]. We demonstrated that overexpression of *KRAS(G12V)* in *KRAS* wild-type cells leads to YB-1 S102 phosphorylation that is dependent on the MAPK and PI3K/Akt pathways [19]. Donaubauer and Hunzicker-Dunn reported that phosphorylation of YB-1 at S102 via ERK/RSK-2 but not PI3K was necessary for follicle-stimulating hormone-mediated expression of target genes required for maturation of follicles towards a preovulatory phenotype [21]. In conflict with this report, YB-1 has been reported to be a substrate for Akt [22,23]. It has been shown that Akt-mediated phosphorylation disables the inhibitory activity of YB-1, thereby enhancing the translation of transcripts involved in oncogenesis [24]. Therefore, the role of PI3K/Akt activity in phosphorylation of YB-1 in *KRAS(G13D)*-mutated cells is still unclear. Phosphatase and tensin homolog deleted on chromosome ten (*PTEN*) is a tumor suppressor gene and one of the most frequently mutated genes in a variety of human tumors, including those of the breast [25,26,27]. Thus far, more than 2700 mutations in *PTEN* in 28 different tumor types have been found [25]. Genetic alterations in *PTEN* lead to deregulation of protein synthesis, cell cycle, migration, growth, DNA repair, and survival signaling [25]. A loss of PTEN function as an antagonist of PI3K and mutation in *PIK3CA* results in deregulation of PI3K signaling, leading to activation of Akt.

In the present study, we investigated the individual role of the MAPK/ERK pathway and the PI3K/Akt pathway in YB-1 S102 phosphorylation in TNBC cells expressing, either *KRAS(G13D)* mutation (MDA-MB-231) cells or *PIK3CA(H1047R)*/*PTEN(E307K)* mutations (MDA-MB-453) [28,29,30]. YB-1 *phosphorylation* was found to be differentially regulated in both cell lines. In *KRAS*-mutated cells the MAPK pathway was the major pathway regulating YB-1 phosphorylation, while in *PIK3CA*/*PTEN*-mutated cells, the PI3K pathway was the dominant pathway regulating YB-1 S102 phosphorylation. We found that dual targeting of PI3K and MEK was found to be an efficient approach to block YB-1 phosphorylation at S102, as well as proliferation of either *KRAS* mutated or *PIK3CA*/*PTEN* mutated cells. Finally, we found that YB-1 has a crucial role in tumor growth of *KRAS*-mutated TNBC cells in vivo.

## 2. Results

### 2.1. YBX1 Knockout Hampers Clonogenic Activity, Inhibits Tumor Growth, and Abrogates the Anti-Proliferative Effect of PI3K and MEK Inhibitors

It is known that YB-1 regulates expression of EGFR and HER2 [31,32]. Likewise, expression levels of total YB-1 protein correlate with poorer survival outcome. In this study, we investigated the level of phosphorylation of YB-1 (S102), phosphorylation of ERK1/2 (T202/Y204), and expression of EGFR in a limited number of tumor tissues from breast cancer patients, including those with TNBC. To this end, we utilized samples from patients undergoing surgery, excluding samples from patients with neoadjuvant chemo-/radiotherapy. Immunofluorescence analysis revealed that the level of YB-1 phosphorylation correlated with enhanced phosphorylation of ERK1/2 and the expression of EGFR in tumor tissues obtained from all of the 6 patients analyzed (Figure S1A–E). Our access to normal tissue was limited to only one patient (PT1). Interestingly, the staining intensity of P-YB-1, P-ERK1/2, and EGFR in the normal tissue was markedly less than in the corresponding tumor tissue obtained from PT1 (Figure S1A,C–E). Staining against total YB-1 protein revealed that the expression level of YB-1 is enhanced in tumor tissues as well (Figure S1F). According to the molecular pathology data summarized in Figure S1B, two out of six patients (PT5 and PT6) were categorized as TNBC patients and four as non-TNBC patients. Overall, however, a positive correlation was observed between the expression of EGFR and the phosphorylation of ERK1/2 and YB-1. This conclusion is further supported by the data obtained from the normal tissue of PT1 and from the tumor tissue of PT3. In both cases, lower EGFR expression was associated with low levels of YB-1 and ERK1/2 phosphorylation (Figure S1C–E).

**Figure 1:**
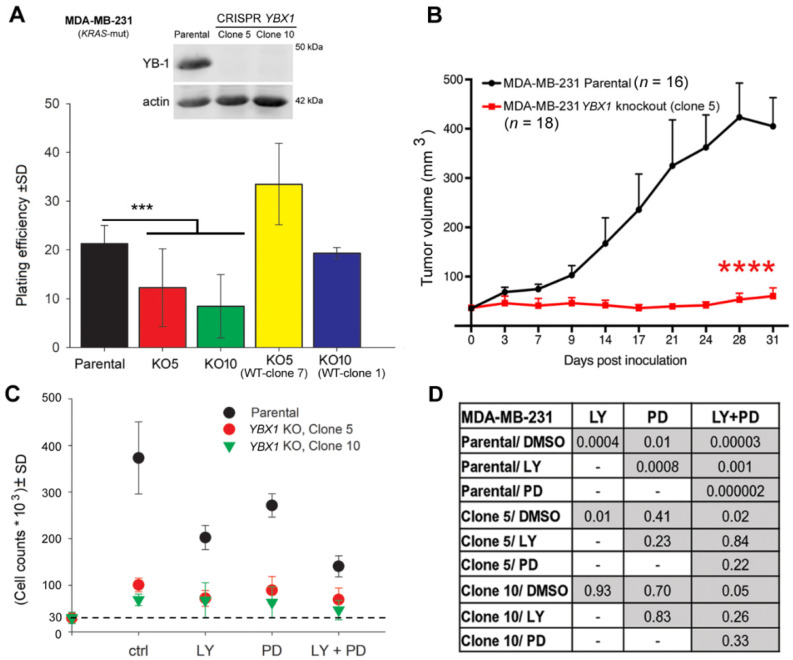
*YBX1* knockout hampers clonogenic activity, inhibits tumor growth, and abrogates the anti-proliferative effect of PI3K and MEK inhibitors. (**A**) The clonogenic activity of the indicated cells with differential YB-1 status, i.e., MDA-MB-231 parental cell, *YBX1* knockout clones 5 and 10, *YBX1* knockout clone 5 after reconstitution of WT YB-1 clone 7 (KO5-WT-Clone 7), and *YBX1* knockout clone 10 after reconstitution of WT YB-1 clone 1 (KO10-WT-Clone 1), was tested using a standard clonogenic assay, and mean plating efficiency was calculated and graphed (parental and *YBX1* knockout clone 5: *n* = 24, 4 experiments; *YBX1* knockout clone 10: *n* = 18, 3 experiments; KO5-WT-Clone 1: *n* = 12, 2 experiments; KO10-WT-Clone 1: *n* = 6, 1 experiment). Western blot data show knockout of *YBX1* in clones 5 and 10. (*** *p* < 0.001, Student’s *t*-test) (**B**) Nude mice were injected with the indicated cells (2 × 10^6^ cells) in both dorsal flanks, and a tumor growth assay was performed. The data are presented as the mean tumor volume ± standard error of the mean (SEM) of the indicated number of tumors after injection of parental MDA-MB-231 cells, compared with MDA-MB-231 *YBX1* knockout cells (clone 5). Asterisks indicate a significant tumor growth delay induced by knockout of *YBX1* (**** *p* < 0.0001, Mann-Whitney U test). (**C**) Cells (3 × 10^4^) were plated in 6-cm culture dishes and treated with inhibitors after 24 h. Cells were counted 5 days after treatment and the results were graphed. The diagram shows the mean number of cells ± standard deviation (SD) (6 data points, 2 independent experiments). The dash line indicates the level of the initial cell count. (**D**) Statistical analyses table shows the degree of significance of the anti-proliferative effect of the indicated inhibitors in MDA-MB-231 parental cells and in *YBX1* knockout clones (Student’s *t*-test).

Knockdown of YB-1, e.g., by siRNA, attenuates the proliferation of tumor cells from different entities [33,34,35]. However, it is unknown to what degree YB-1 is involved in tumor cell proliferation. Here, after *YBX1* knockout by CRISPR/Cas9, we tested the absolute effect of YB-1 on cell proliferation, clonogenic activity, and tumor growth. Western blot data confirmed the knockout of *YBX1* in two representative clones (clone 5 and clone 10) of MDA-MB-231 cells (Figure 1A, Figure S2A). In both clones tested, *YBX1* knockout did strongly but not completely inhibit clonogenic activity (Figure 1A). An approximately 50–60% inhibition of plating efficiency was achieved in both clones (Figure 1A). To further assess the role of YB-1 in clonogenic activity, we reintroduced wild-type YB-1 in *YBX1* knockout MDA-MB-231 cells (clone 5 and clone 10) (Figure S2B,C) and tested the clonogenic activity. The data shown in Figure 1A indicate that reintroduction of YB-1 in *YBX1* knockout clones restored clonogenic activity to the level observed in parental cells (Figure 1A). We further tested the role of YB-1 in tumor growth in a xenograft model in vivo. In line with the role of YB-1 in clonogenic activity (Figure 1A), *YBX1* knockout inhibited tumor growth after inoculation with MDA-MB-231 cells throughout the 30 days of observation (Figure 1B). The data presented in Figure 1C indicates that, similar to the effect on clonogenic inhibition, *YBX1* knockout did not completely inhibit cell proliferation. It inhibited proliferation of MDA-MB-231 cells in both clones by about 77%. The same effect was achieved by dual targeting of PI3K and MEK (Figure 1C). Dual targeting of the PI3K and MAPK pathways has been suggested before [36,37], through a synthetic lethal interaction between the two pathways [38]. Since the role of YB-1 in the described interaction between the two pathways has not been reported, here we tested the antiproliferative effect of the inhibitors of PI3K and MEK in MDA-MB-231 parental and *YBX1* knockout cells. The data shown in Figure 1C indicates that treatment with the PI3K inhibitor LY294002 (LY) or the MEK inhibitor PD98059 (PD) significantly inhibited proliferation of parental cells compared with DMSO-treated cells. Moreover, the combination of both inhibitors on cell proliferation in parental cells was much stronger than single treatments (Figure 1D) and synergistic, tested by applying the fractional product method [39,40]. In *YBX1* knockout cells (clone 5), a very slight but significant anti-proliferative effect was observed after treatment with LY (*p* = 0.01) and the combination of LY and PD (*p* = 0.02). The combination of LY + PD did not show any significant difference in proliferation inhibition compared with the LY (*p* = 0.84) or PD treatment (*p* = 0.22). In *YBX1* knockout clone 10, single as well as dual treatment with the inhibitors had no significant effect on cell proliferation.

### 2.2. S102 Phosphorylation of YB-1 Is Differentially Affected by the MAPK and PI3K Pathways in KRAS Mutated and PIK3CA/PTEN Mutated Breast Cancer Cells

**Figure 2:**
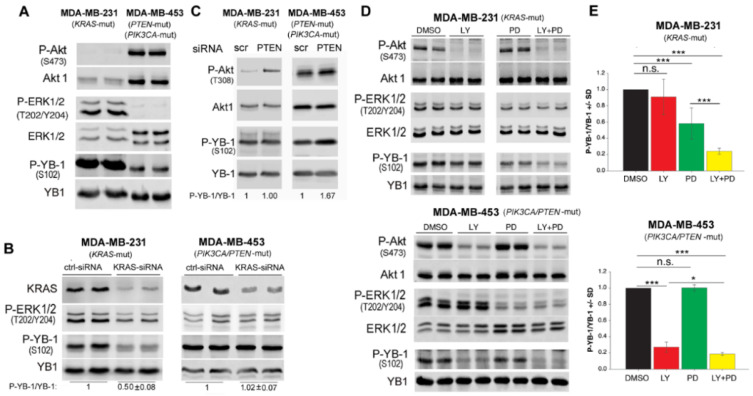
S102 phosphorylation of YB-1 is differentially affected by the MAPK pathway and PI3K in *KRAS*-mutated MDA-MB-231 and *PIK3CA*/*PTEN*-mutated MDA-MB-453cells. (**A**) Protein samples from 2 parallel cultures were isolated, and the basal phosphorylation levels of AKT, ERK1/2 and YB-1 were detected by Western blotting. Blots were stripped and incubated with antibodies against total proteins. (**B**,**C**) Cells were transfected with control-siRNA (ctrl-siRNA), *KRAS*-siRNA or *PTEN*-siRNA. Protein samples were isolated at seventy-two hours after transfection (**B**) and 48 h after transfection (**C**). The indicated phospho- as well as total proteins were detected by Western blotting. The densitometry analysis shows the mean ratio of P-YB-1/YB-1 ± SD (*n* = 4 data from 2 independent experiments) (**B**), normalized to 1 in ctrl-siRNA conditions (**B**,**C**). (**D**) Indicated cells were treated with the PI3K inhibitor LY294002 (LY, 10 μM), MEK inhibitor PD98059 (PD, 20 μM), or a combination of LY and PD for 2 h and protein samples were isolated. Protein samples exposed to the same treatment conditions were isolated from 2 parallel cultures. The indicated phospho- as well as total proteins were detected by Western blotting. (**E**) Densitometry values represent the mean P-YB-1/YB-1 ratio normalized to 1 in the DMSO-treated condition (MDA-MB-231: *n* = 6 data from 3 independent experiments; MDA-MB-453: *n* = 4 data from 3 independent experiments). (* *p* < 0.05, *** *p* < 0.001, Student’s *t*-test), n.s.: not significant.

As supported by the data presented in Figure 2A, *KRAS* mutation in MDA-MB-231 cells and *PIK3CA*/*PTEN* mutation in MDA-MB-453 cells [30] leads to the activation of the MAPK and PI3K pathways, respectively. Phosphorylation of YB-1 in MDA-MB-231 was markedly stronger than in MDA-MB-453 cells. However, this difference was not as strong as the difference in phosphorylated ERK1/2. This data indicates that *KRAS* mutation is most likely the key component involved in phosphorylation of YB-1 in MDA-MB-231 cells. This was also confirmed after knockdown of KRAS by siRNA in both cell lines. As shown in Figure 2B, knockdown of KRAS in MDA-MB-231 cells inhibited YB-1 phosphorylation (Figure 2B). In contrast, no effect of *KRAS* knockdown on YB-1 phosphorylation was observed in MDA-MB-453 cells. Based on this data we concluded that MDA-MB-231 cells are addicted to the KRAS-regulated MAPK-dependent YB-1 phosphorylation. In contrast, YB-1 phosphorylation in MDA-MB-453 cells may depend on the PTEN-dependent PI3K/Akt pathway. To test this hypothesis, we transfected both cell lines with PTEN-siRNA and analyzed phosphorylated Akt (T308) as an indication for successful knockdown of PTEN. As shown in Figure 2C, enhanced Akt phosphorylation in both cell lines 48 h after siRNA transfection was associated with an approximately 70% increase in YB-1 phosphorylation in MDA-MB-453 cells but not in MDA-MB-2321 cells. This finding shows the suppression of the function of PI3K/Akt on YB-1 phosphorylation in *KRAS*-mutated cells. According to this data, we concluded that MDA-MB-453 cells may express *KRAS* wild-type. In our literature search, we found two papers that indicated a *KRAS* point mutation in this cell line [41,42]. However, a direct comparison to MDA-MB-231 cells, MDA-MB-453 cells presented extremely low levels of ERK1/2 phosphorylation (Figure 2A) that was unaffected by *KRAS*-siRNA (Figure 2B). To confirm the MDA-MB-453 cells harbor *KRAS* wild-type, we performed next generation sequencing in authenticated cell. The results indicated that that MDA-MB-453 cells harbor both *KRAS* and *HRAS* wild-type.

Next, we tested the effectiveness of single PI3K and MEK targeting as well as that of dual targeting on YB-1 phosphorylation in *KRAS(G13D)*-mutated MDA-MB-231 and *PTEN* mutated MDA-MB-453 cells (Figure 2D). Interestingly, inhibition of MEK but not PI3K significantly and strongly inhibited S102 phosphorylation of YB-1 in MDA-MB-231 cells (Figure 2D). In *PTEN*-mutated MDA-MB-453 cells, targeting PI3K by LY strongly inhibited YB-1 phosphorylation at S102 while MEK inhibition by PD did not affect the YB-1 phosphorylation status (Figure 2D). Independent of the individual dominant pathway regulating YB-1 phosphorylation, dual targeting of MEK and PI3K efficiently inhibited YB-1 phosphorylation in both cell lines when compared to the effect of the appropriate targeting approach, i.e., MEK inhibition in MDA-MB-231 cells and PI3K inhibition in MDA-MB-453 cells (Figure 2D). The densitometry data is presented in Figure 2E.

The major role of PI3K in YB-1 phosphorylation in cells expressing *PTEN* mutations was also tested in the *PTEN-/-* prostate cancer cell line PC3 [43]. Treatment with LY strongly inhibits phosphorylation of YB-1 up to 48 h (Figure S3).

### 2.3. Akt Differentially Affects S102 Phosphorylation of YB-1 in PTEN Wild-Type vs. PTEN Mutated Cells

**Figure 3:**
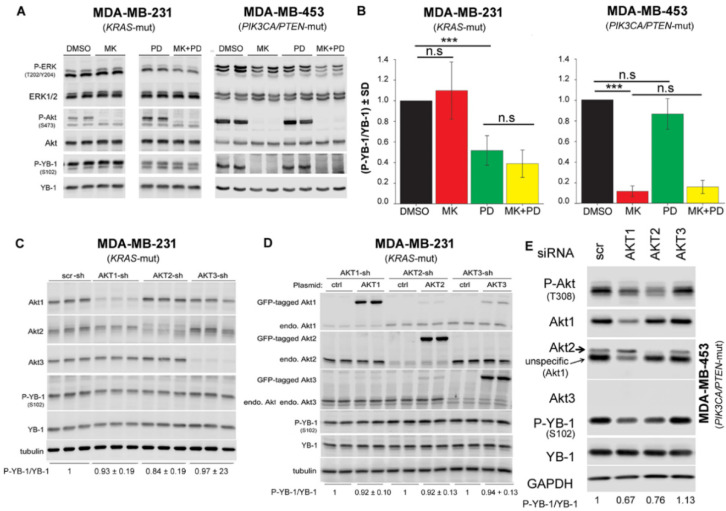
Akt differentially affects S102 phosphorylation of YB-1 in *PTEN* wild-type vs. *PTEN* mutated cells. (**A**) The indicated cells were treated with the Akt inhibitor MK2206 (MK, 10 μM), PD98059 (PD, 20 μM), or a combination of MK and PD for 2 h. Control cells received DMSO. Protein samples were isolated, and the indicated proteins were detected by Western blotting. Protein samples exposed to the same treatment conditions were isolated from 2 parallel cultures, and the results are shown in the panel. (**B**) Densitometry values represent the mean P-YB-1/YB-1 ratio normalized to 1 in DMSO treated condition (MDA-MB-231: *n* = 7 data from 3 independent experiments; MDA-MB-453: *n* = 4 data from 3 independent experiments). (*** *p* < 0.001, Student’s *t*-test), n.s.: not significant. (**C**) Protein samples were isolated from MDA-MB-231 cells stably transfected with scrambled-shRNA (scr-sh), Akt1-shRNA, Akt2-shRNA, or Akt3-shRNA. Protein samples were isolated from three parallel cultures. (**D**) MDA-MB-231 cells stably expressing shRNA against endogenous Akt1, Akt2, or Akt3 were transiently transfected with plasmid expressing eGFP-tagged-Akt1, -Akt2, or -Akt3. Forty-eight hours after transfection, protein samples were isolated from two parallel cultures. (**E**) Cells were transfected with 50 nM of indicated siRNA, and protein samples were isolated after 48 h. Levels of phosphorylation and expression of indicated proteins were analyzed by Western blotting. Following detection of phospho-proteins, blots were stripped and re-incubated to detect total proteins. GAPDH was used as loading control.

Data presented in Figure 2D,E indicates that S102 phosphorylation of YB-1 depends on PI3K activity in PIK3CA/*PTEN*-mutated MDA-MB-453 cells but not in *KRAS*-mutated MDA-MB-231 cells. As Akt is the major substrate of PI3K, we investigated whether targeting Akt differentially affects YB-1 phosphorylation in the two cell lines tested. Likewise, we tested whether the combination of the Akt inhibitor MK-2206 (MK) and MEK inhibitor PD is as effective as the combination the PI3K inhibitor LY and PD on inhibition of YB-1 phosphorylation, shown in Figure 2D,E. The data presented in Figure 3A indicates that inhibition of Akt through pretreatment with MK did not affect YB-1 phosphorylation at S102 in MDA-MB-231 cells. In contrast, MK completely blocked YB-1 phosphorylation in MDA-MB-453 cells. Similar to the data presented in Figure 2A, PD significantly reduced YB-1 phosphorylation in MDA-MB-231, but not in MDA-MB-453 cells (Figure 3A,B). The combination of MK and PD did not have an additional inhibitory effect on YB-1 phosphorylation, compared to PD alone in MDA-MB-231, or compared to MK alone in MDA-MB-453 cells. These data indicate that the effect of PI3K inhibition on YB-1 S102 phosphorylation in *KRAS*-mutated MDA-MB-231 is Akt independent. Next, we specifically investigated the role of Akt isoforms on YB-1 phosphorylation in MDA-MB-231 and MDA-MB-453 cells using a genetic approach. In MDA-MB-231 cells, each Akt isoform was stably knocked down with shRNA, as reported previously [44]. The Western blot and related densitometry values presented in Figure 3C indicate that the level of P-YB-1 was not significantly affected after knockdown of any of the Akt isoforms. To determine whether YB-1 S102 phosphorylation could be restored, we overexpressed each Akt isoform in corresponding Akt isoform knockdown MDA-MB-231. Analysis of the phosphorylation status of YB-1 showed that overexpression of any AKT isoforms did not rescued YB-1 phosphorylation in MDA-MB-231 cells (Figure 3D). Additionally, the role of Akt isoforms on YB-1 phosphorylation was investigated after single knockdown, double knockdown, or triple knockdown of the three Akt isoforms. None of these conditions inhibited phosphorylation of YB-1 (Figure S4). In contrast to the effect on MDA-MB-231 cells, knockdown of Akt1 and Akt2 by siRNA in MDA-MB-453 inhibited YB-1 phosphorylation by about 35% and 25%, respectively. Western blotting indicated that MDA-MB-453 cells did not express Akt3. Therefore, Akt3-siRNA did not affect YB-1 phosphorylation in this cell line (Figure 3E).

### 2.4. Dual Targeting of PI3K and MEK Is an Efficient Approach to Block Proliferation of RAS-Mutated and PIK3CA/PTEN-Mutated TNBC Cells in a YB-1-Dependent Manner

**Figure 4:**
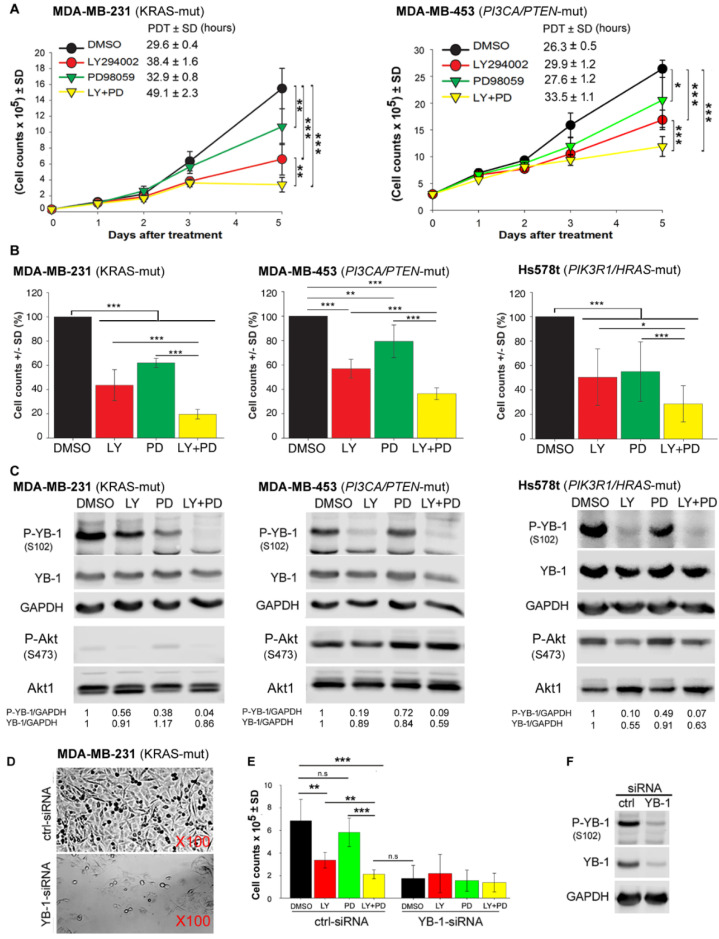
Dual targeting of PI3K and MEK is an efficient approach to block proliferation of *KRAS*-mutated TNBC cells (MDA-MB-231), *PIK3CA*/*PTEN*-mutated TNBC cells (MDA-MB-453), and *PIK3R1/HRAS*-mutated TNBC (Hs578t) cells in a YB-1-dependent manner. (**A**) A proliferation assay was performed after cell treatment with the PI3K inhibitor LY (10 μM), MEK inhibitor PD (20 μM), or a combination of both inhibitors in 6-cm plates (30 × 10^3^ cells for MDA-MB-231 and 300 × 10^3^ cells for MDA-MB-453). The concentration of vehicle (DMSO) was similar under all conditions, including the control (0.2%). Asterisks indicate significant differences in PDT between the indicated treatment conditions (* *p* < 0.05, ** *p* < 0.01, and *** *p* < 0.001, Student’s *t*-test; *n* = 6 data points, 2 experiments). (**B**) Cells were counted in the proliferation assay on day 5 after indicated treatments, mean percentage of cells was calculated for each inhibitor-treated condition and normalized to the DMSO-treated condition. Bar graphs present the mean of 9 data points from 3 independent experiments in MDA-MB-231 and MDA-MB-453 cells and 7 data points from 3 independent experiments in Hs578t cells. (* *p* < 0.05, ** *p* < 0.01, and *** *p* < 0.001, Student’s *t*-test) (**C**) Cells were treated as indicated, protein samples were isolated on day 5 after treatment, and the levels of indicated proteins were detected by Western blotting. Densitometry values represent the P-YB-1/GAPDH, YB-1/GAPDH ratios normalized to 1 in the DMSO-treated control. GAPDH was detected as the loading control. Experiments were repeated 3 times in MDA-MB-231 and MDA-MB-453 cells and 2 times in Hs578t cells. (**D**) Representative light microscopy images of cells transfected with control (ctrl)-siRNA and YB-1-siRNA on day 6 after transfection. (**E**) MDA-MB-231(G13D) cells were transfected with 50 nM control (ctrl) siRNA or YB-1-siRNA, and 24 h after transfection, the cells were treated with the indicated inhibitors. Thereafter, the cells were counted on day 6, and the results were graphed. Bar graphs present the mean cell count of 6 parallel cultures obtained from 2 independent experiments. Asterisks indicate significant differences between the indicated treatment conditions (* *p* < 0.05, ** *p* < 0.01, and *** *p* < 0.001, Student’s *t*-test), n.s.: not significant. (**F**) Protein samples were isolated from the DMSO-treated cells, and the level of YB-1 knockdown by siRNA was confirmed by Western blotting.

So far, we showed that, independent of the individual dominant pathway regulating YB-1 phosphorylation, dual targeting of MEK and PI3K efficiently inhibited YB-1 activity in *KRAS*-mutated as well as *PIK3CA/PTEN*-mutated TNBC cells (Figure 2). In further experiments, we investigated whether this approach would effectively inhibit cell proliferation. As shown in Figure 4A and reflected by the population doubling time (PDT), dual targeting of PI3K and MEK significantly prolongs the doubling time in both *KRAS*-mutated and *PIK3CA/PTEN*-mutated cell lines. Furthermore, the anti-proliferative effect of the combination of PI3K and MEK inhibitors was significantly stronger that effect of each inhibitor alone not only in *KRAS*-mutated and *PIK3CA/PTEN*-mutated cell lines but also in the TNBC cell line Hs578t that is known to harbor both a *PIK3R1* and *HRAS* mutation [45] (Figure 4B). The effect of dual targeting on YB-1 phosphorylation was much stronger than the effect of single treatment 5 days after treatment in the three cell lines tested in this study (Figure 4C). In line with the data shown in Figure 2A, simultaneous detection of P-Akt (S473) in MDA-MB-231 and MDA-MB-453 revealed a very strong phosphorylation in MDA-MB-453 cells. Interestingly, long-term inhibition of either PI3K or MEK led to the reactivation of Akt at S473. Inhibition of YB-1 phosphorylation by either of the inhibitors or the combination of both inhibitors was not correlated with the phosphorylation status of Akt at S473. In MDA-MB-231 cells, an efficient inhibition of YB-1 phosphorylation by MEK inhibitor PD was associated with stimulated phosphorylation of Akt at S473. By adding siRNA against *KRAS* we could show that the Akt reactivation depends on *KRAS* expression and occurs through PI3K (Figure S5). In MDA-MB-453 and Hs578t cells, where a strong effect of the PI3K inhibitor LY on YB-1 phosphorylation is observed, no effect on Akt phosphorylation (in MDA-MB-453 cells) or a very slight effect (in Hs578t cells) was observed (Figure 4C). A marked correlation between inhibition of cell proliferation (Figure 4B) and attenuating YB-1 phosphorylation (Figure 4C) by the inhibitors was observed in all cell lines tested.

Importantly, and similar to the data obtained in *YBX1* knockout cells (Figure 1), YB-1 knockdown in MDA-MB-231 cells (Figure 4F) inhibited cell proliferation to the same degree that was observed by dual targeting of MEK and PI3K (Figure 4D,E). Cell proliferation was not affected further after single or dual treatment in YB-1 knockdown cells (Figure 4E). The uncropped Western blots presented throughout the manuscript have been shown in Figure S6.

## 3. Discussion

In the present study, we showed that S102 phosphorylation of YB-1 in three TNBC cell lines depends on mutation status of the components of the MAPK/ERK and PI3K/Akt pathways. In *KRAS*-mutated cells, YB-1 S102 phosphorylation is mainly dependent on the MAPK/ERK pathway while in *PIK3CA*/*PTEN*-mutated cells, it is entirely dependent on the PI3K/Akt pathway. Independent of the dominant pathway regulating YB-1 phosphorylation, dual targeting of MEK and PI3K, but not of MEK and Akt was found to be the most effective approach to block YB-1 phosphorylation and to inhibit YB-1 dependent cell proliferation. Finally, *YBX1* knockout was shown to be sufficient to block TNBC tumor growth.

According to publicly available next-generation sequencing studies, i.e., the Cancer Cell Line Encyclopedia from the Broad Institute (https://portals.broadinstitute.org/ccle), and our own NGS data for *HRAS* and *KRAS* in MDA-MB-453 cells, the three TNBC cell lines used in the present study harbor different mutations in the components of the PI3K/Akt and MAPK/ERK pathways. MDA-MB-231 cells express *KRAS(G13D)* and *BRAF(G464V)* mutations that lead to the hyperactivation of the MAPK/ERK pathway in association with suppression of Akt phosphorylation. MDA-MB-453 cells have a hotspot *PIK3CA(H1047R)* mutation that leads to constitutive PI3K/Akt activity [28,29] as well as a *PTEN(E307K)* mutation [30]. In contrast to these two cell lines, Hs578t cells harbor an in-frame insertion mutation (453_454insN) in *PIK3R1*, encoding PI3K regulatory subunit 1 whose impact on PI3K/Akt activity is unknown, and an *HRAS(G12D)* mutation that leads to the hyperactivation of both the PI3K/Akt and MAPK/ERK pathways. In the cell lines tested, pretreatment with the pharmacological inhibitors or genetic approaches led to differential effects on phosphorylation of YB-1 at S102, which was linked to the hyperactivation of each pathway through the mutation in the components of the specific pathway. The S102 phosphorylation of YB-1 in MDA-MB-231 and MDA-MB-453 cells was dependent on the MAPK/ERK, and PI3K/AKT pathways, respectively, as shown after short-term treatment with the inhibitors of the respective pathway. In Hs578t cells, phosphorylation of YB-1 was mainly dependent on the PI3K/Akt pathway.

Since crosstalk and compensatory mechanisms exist between the PI3K/Akt and MAPK/ERK pathways, especially in tumor cells [46,47], the effect of the inhibitors after a short-term treatment may not reflect the ultimate effect that is achieved by long-term treatment. This statement is supported by the data presented in Figure 2B and Figure 4C, in which the inhibitory effect of the PI3K inhibitor on YB-1 phosphorylation was enhanced in *KRAS(G13D)*-mutated MDA-MB-231 cells after long-term treatment (about 40% inhibition after 5 days of treatment vs. 10% inhibition after 2 h of treatment). Additionally, in *PIK3CA*/*PTEN*-mutated MDA-MB-453 cells, the inhibitory effect of MEK inhibitor on YB-1 phosphorylation increased to about 28% inhibition after 5 days of treatment versus no effect after 2 h of treatment.

The changes in the pattern of the effect on YB-1 phosphorylation after long-term targeting could be due to the crosstalk between the PI3K/Akt and MAPK/ERK pathways. There were a few reports that demonstrated a beneficial effect of targeting both pathways [36,37] and a synthetic lethal interaction between the two pathways [38]. In the present study, we show that the long-term inhibition of MEK leads to the enhanced phosphorylation of Akt through a PI3K-dependent pathway mainly in *KRAS*-mutated MDA-MB-231 cells and also to a lesser degree in other cell lines tested in the study. Knockdown of KRAS in *KRAS(G13D)*-mutated cells led to the PI3K-dependent reactivation of Akt as well (Figure S5). Thus, MEK inhibition can mimic *PIK3CA/PTEN* mutation and leads to PI3K activity in *PIK3CA*/*PTEN* wild-type MDA-MB-231 cells.

Importantly, dual targeting of PI3K and MEK or single knockout of YB-1 suppresses cell proliferation in *KRAS*-mutated MDA-MB-231 cells by about 70–80%. Similar effects are achieved in terms of inhibition of clonogenic activity in *YBX1* knockout clones. This data may indicate that cell proliferation and clonogenicity is partially regulated through a pathway that is independent of YB-1 and is therefore unaffected by targeting PI3K and MEK. Alternatively, deregulation of non-coding micro-RNA (miRNA) expression can play an important role in variety of cellular functions, i.e., cell proliferation and survival. Molecular targeting can change miRNA expression and consequently the effectiveness of the individual targeting approach. In this regard, accumulating evidence indicates a novel role of YB-1 in miRNA processing, e.g., downregulation of miR-29b [48] or induction of miR-155 [49]. Thus, according to this novel function of YB-1, it can be expected that *YBX1* knockout or the dual-targeting approach can potentially lead to dysregulation of miRNA that diminishes the effectiveness of each approach, in terms of inhibiting cell proliferation and clonogenicity. Additionally, analysis of the protein expression profile revealed that the chloride intracellular channel 4 protein, a protein involved in endothelial cell proliferation [50], is upregulated in *YB-1* knockout clones (clones 5 and 10) compared to the parental cells (our unpublished data). In this context, our data from animal studies resulted in superior growth delay of *YB-1* knockout cells after 31 days. This may be because of the effect of YB-1 on tumor development, microenvironment, migration, etc. Since occurrence of these cancer hallmarks is proliferation-independent, absence of YB-1 can potentially suppress either of these hallmarks leading to the suppression of tumor growth, even if cells can partially proliferate. In this context, a very recent report by Kosnopfel et al. showed that the extracellular YB-1 exerts a stimulating effect on melanoma cell migration, invasion, and tumorigenicity [51].

**Figure 5:**
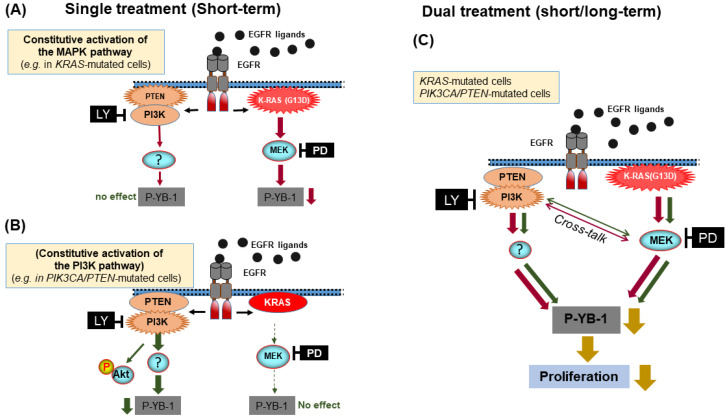
Model demonstrating dual targeting of PI3K and MEK as an efficient approach to target constitutive activation of YB-1 in *KRAS*(G13D)-mutated and *PIK3CA/PTEN*-mutated TNBC cells. A short-term (2 h) inhibition of constitutive PI3K or (**A**) MEK activity (**B**) efficiently inhibits YB-1 phosphorylation at S102, while long-term inhibition of each pathway leads to YB-1 reactivation by the alternative pathway (**C**). Dual targeting of both pathways blocks the crosstalk-mediated reactivation of YB-1, leading to the efficient blockage of cell proliferation.

The Akt family includes three isoforms and is one of the major PI3K substrates. By directly targeting Akt using the Akt inhibitor MK-2206 as well as genetic approaches, it was shown that phosphorylation of YB-1 at S102 is Akt independent in *KRAS*-mutated MDA-MB-231 cells. In *PI3K*/*PTEN*-mutated MDA-MB-453 cells, knockdown of Akt1 and Akt2 partially inhibited YB-1 phosphorylation. Since this cell line did not express Akt3 (Western blotting data), no effect on YB-1 phosphorylation was observed after Akt3 knockdown. Akt-dependent YB-1 phosphorylation in MDA-MB-231 cells was shown by MK. Although a cell line-dependent function of PTEN and Akt on YB-1 phosphorylation was shown in the present study, it is important to note that the P-Akt(S473) status following long-term treatment with the inhibitors does not predict effectiveness of these inhibitors on YB-1 phosphorylation (Figure 4C). Together, these data indicate that in contrast to the proposed major role of RSK in YB-1 phosphorylation [14,15], phosphorylation of YB-1 at S102 is dependent on mutation status of the components of the PI3K/Akt and MAPK/ERK pathway (Figure 5A,B). In this context, hyperactivation of the PI3K/Akt pathway can shift the MAPK/ERK dependency of YB-1 phosphorylation to PI3K/Akt dependency (Figure 5B). Therefore, our data uncover an interaction between the two pathways affecting YB-1 activity and, under this condition, dual targeting of PI3K and MEK becomes an efficient approach to block proliferation as depicted in Figure 5C. According to this model, RSK targeting cannot be used as an effective approach to target YB-1, a conclusion that is also supported by the study in colorectal cancer cells using RSK inhibitor LJI308 [52].

In a limited number of tissues from breast cancer patients, we also studied the status of P-YB-1 and P-ERK1/2 as well as expression of YB-1 in the samples for which the corresponding normal tissue was available. Although, analyzing the mutation status of the components of the PI3K/Akt and MAPK/ERK pathways in the tumor tissues under this study was not plausible, the hyperphosphorylation of YB-1 and of ERK1/2 in 5 out of 6 ductal carcinoma tissues was observed. Interestingly, the described correlation was also observed in tumor tissue from patient number 3 (PT3) in which the level of P-YB-1 was also low in association with lower levels of P-ERK1/2. According to the histopathology data, two out of six tumors analyzed in our study are TNBC, which also presented hyperactivation of the ERK/YB-1 pathway. Hyperactivation of ERK1/2 and YB-1 in non-TNBC tumors might be due to other alterations and mutations in the components of the MAPK/ERK and PI3K/AKT pathways. Enhanced phosphorylation of YB-1 and ERK1/2 was also associated with enhanced EGFR expression. This is in line with previous studies that reported a link between expression of YB-1 and EGFR in breast cancer patients [32,53,54]. Independent of the mechanism(s) involved in the activation of the YB-1 in non-TNBC cells, this observation additionally highlights the importance of our findings in targeting the YB-1 pathway not only in TNBC but also in non-TNBC. Additionally, the low level of phosphorylated YB-1 in normal tissue obtained from PT1 ex vivo and previously published data in vitro [19], as well as in colorectal cancer tissues [52] suggest that the proposed targeting strategy in our study might spare the normal tissue and thus generate a therapeutic window of opportunity for cancer treatment. The focus of our study was uncovering the signaling pathways involved in YB-1 S102 phosphorylation, but it is known that YB-1 is phosphorylated on other residues as well. In this context, phosphorylation of YB-1 at S165 and S176 and the impact of S165 phosphorylation on cell proliferation and tumorigenicity have been reported [55,56]. It remains to be investigated to what degree phosphorylation of these residues is influenced by targeting either of the PI3K/Akt and RAS/MAPK pathways or after dual targeting of both pathways.

Together, dual inhibition of MAPK and PI3K pathways was not successful in clinic due to toxicity issues. Likewise, YB-1 inhibition by specific inhibitors is not feasible at present. Recently, Goulielmaki and colleagues reported that the novel dual bioactive compounds, e.g., DPS-2 targets both MEK and PI3K and showed potent anticancer effects [57]. Such compounds might have limited toxicity, compared to combining the inhibitors of MEK and PI3K. An alternative approach would be applying naturally occurring flavonoid, Fisetin, whose inhibitory effect on YB-1 has been described in different tumor entities [58]. These approaches that effectively and simultaneously inhibit MAPK and PI3K pathways might be effective to block YB-1 signaling in other frequently *KRAS*-mutated tumor entities, i.e., pancreatic, colorectal and lung cancers, as well as in those tumor entities with constitutive activation of the PI3K/Akt pathway.

## 4. Materials and Methods

### 4.1. Cell Lines, Antibodies, and Reagents

MDA-MB-231 (ATCC^®^ HTB-26™), MDA-MB-453 (ATCC^®^ HTB-131™), and Hs578t (ATCC^®^ HTB-126™) cells were used. The identity of cells was confirmed (Multiplexion GmbH, Heidelberg, Germany). MDA-MB-453 cells were kindly provided by Dr. Andre Koch (Department of Women’s Health, Research Institute for Women’s Health, University Hospital Tuebingen, Tuebingen, Germany). MDA-MB-231 cells stably transfected with scrambled-shRNA, Akt1-shRNA, Akt2-shRNA and Akt3-shRNA that were kindly provided by Dr. Manfred Jücker, Institute of Biochemistry and Signal Transduction, University Medical Center Hamburg-Eppendorf, Hamburg, Germany. Cells were cultured in DMEM supplemented with 10% fetal calf serum (FCS) and 1% penicillin-streptomycin. Cells were incubated in a humidified atmosphere of 93% air and 7% CO_2_ at 37 °C.

Antibodies against phospho-YB-1 (S102) (Cat.#2900), YB-1 (Cat.#4202), phospho-Akt (S473) (Cat.#9271), phospho-ERK1/2 (T202/Y204), (Cat.#4377), phospho-p90RSK (T359/S363) (Cat.#9344S), and ERK1/2 (Cat.#4695) as well as YB-1-siRNA (Cat #6206) were purchased from Cell Signaling (Frankfurt, Germany). An Akt1 antibody (Cat.#610877) was purchased from BD Biosciences (Heidelberg, Germany). A KRAS antibody (Cat.#ab157255) was purchased from Abcam (Cambridge, UK). Actin antibody was purchased from Sigma-Aldrich (Taufkirchen, Germany). The PI3K inhibitor LY294002 (Cat.#440202) was purchased from Calbiochem (Schwalbach, Germany). The MEK inhibitor PD98059 (Cat.#S1177) and Akt inhibitor MK-2206 (Cat.#S1078) were purchased from Selleck Chemicals (Munich, Germany). Small interfering RNAs (siRNAs) against KRAS (Cat.#M-005069) and were purchased along with a nontargeting siRNA (Cat.#D-001810-10) from Thermo Scientific Dharmacon (Bonn, Germany). Lipofectamine 2000, Lipofectamine RNAiMAX, Opti-MEM, and YO-PRO were purchased from Invitrogen (Darmstadt, Germany).

### 4.2. YBX1 Knockout Using CRISPR/Cas9

*YBX1* gene knockout was performed via CRISPR/Cas9-mediated genome engineering as described previously [13] using the lentiCRISPRv2 one vector system harboring *YBX1*-specific sgRNA sequences (YB-1 sgRNA1 (forward) 5′-cac cgg gac cat acc tgc gga atc g-3′, YB-1 sgRNA1 (reverse) 5′-aaa ccg att ccg cag gta tgg tcc c-3′, YB-1 sgRNA2 (forward) 5′-cac cgc gta gtg ccg ggc ttg gtg tcg g-3′, YB-1 sgRNA2 (reverse) 5′-aaa ccc gac acc aag ccc ggc act acg c-3′; Sigma-Aldrich, Taufkirchen, Germany). Following lentiviral transduction and selection with 2 μg/mL puromycin for 14 days, single cell clones were generated and selected based on loss of YB-1 expression.

### 4.3. YB-1 Overexpression

Breast cancer cells with inducible overexpression of 3XFLAG-tagged wild-type YB-1 (YB-1^WT^, cloned into pLVX-Tight-Puro) were generated by lentiviral gene transfer as described by Kosnopfel et al. [7]. Transgene expression was induced by adding 2 μg/mL doxycycline (AppliChem, Darmstadt, Germany) to the culture medium.

### 4.4. Treatment with the Inhibitors

The PI3K inhibitor LY294002 (LY) and Akt inhibitor MK-2206 (MK) were diluted in dimethyl sulfoxide (DMSO), and 10 mM stock solutions were stored at −20 °C. The MEK inhibitor PD98059 (PD) was prepared as a 50 mM stock solution. For treatment, stock solutions of the inhibitors were diluted in culture medium and applied to the cells.

### 4.5. siRNA Transfection

Cells were transfected with 50 nM nontargeting siRNA or specific siRNA using Lipofectamine 2000 transfection reagent according to the protocol of the manufacturer. Twenty-four hours after transfection, the medium was changed, and 48 h later, the protein samples were prepared and subjected to Western blotting. For the proliferation assay, YB-1 knockdown was performed through reverse transfection using RNAiMAX according to the manufacturer’s protocol.

### 4.6. Proliferation Assay

To test the effect of the MEK and PI3K inhibitors alone or in combination on cell proliferation, cells were seeded in 6-cm tissue culture dishes or 6-well plates and treated with the indicated inhibitors after 24 h. At days 1, 2, 3, and 5 after treatment, cells were collected by trypsinization and counted, and growth curves were prepared. The cell population doubling time (PDT) was calculated. Statistical analysis was performed to compare the PDT in the control cells and cells treated with the indicated inhibitors. To analyze the role of YB-1 in the anti-proliferative effect of the PI3K and MEK inhibitors, MDA-MB-231 cells were reverse transfected with control-siRNA or YB-1-siRNA using Lipofectamine RNAiMAX according to the manufacturer’s instructions and cultured in 6-well plates. After 24 h, control- as well as YB-1-siRNA transfected cells were treated with the inhibitors. Cells were trypsinized and counted 5 days after inhibitor treatment. We also tested the antiproliferative effect of the PI3K inhibitor LY, the MEK inhibitor PD, and a combination of both inhibitors in parental MDA-MB-231 cells and in two *YBX1* knockout clones.

### 4.7. Protein Extraction and Western Blotting

After the indicated treatments, total protein lysate was isolated as described previously [59]. Following protein quantification using a Bio-Rad DC protein assay, samples were subjected to sodium dodecyl sulfate polyacrylamide gel electrophoresis (SDS-PAGE), and the expression of specific proteins was assessed by Western blot analysis using specific antibodies.

### 4.8. Clonogenic Assay

Clonogenic assays were performed as previously described [60]. In brief, cells were plated in 6-well plates in medium containing 20% serum. The culture dishes were stained with Coomassie blue after 10 days. Colonies with more than 50 cells were counted, and the plating efficiency (number of colonies/number of seeded cells) was calculated and graphed.

### 4.9. Mouse Xenograft Tumor Growth Assay

Athymic nude mice (4- to 6-week-old females) were obtained from Envigo Laboratories (Indianapolis, IN, USA). All animal procedures and maintenance were conducted in accordance with the institutional guidelines of the University of Wisconsin-Madison. The Ethic code for that study is: UW-M005805. Mice were injected with 2 × 10^6^ cells (MDA-MB-231 parental or MDA-MB-231 *YBX1* knockout clone 5) in both dorsal flanks. Tumor volume measurements were evaluated using digital calipers and calculated twice per week from day 7 after tumor cell injection using the following formula: π/6 × (small diameter)^2^ × large diameter.

### 4.10. Immunofluorescence Staining

Breast cancer patients undergoing surgery as a primary treatment were included in the study. Previous neo-adjuvant treatment or chemoradiotherapy were the exclusion criteria. The study was approved by the Ethics Committee of the Medical Faculty of the University of Tuebingen (confirmation #426/2013BO1). All patients provided signed, informed consent. Freshly excised material from six tumors and one corresponding normal tissue were retrieved during surgery and embedded into paraffin as previously described [61]. Cross-sections of the paraffin-embedded material were used for staining. Paraffin sections were deparaffinized and rehydrated. Slides were incubated in citrate buffer at pH 6.0 (Thermo Scientific) using a pressure cooker. Sections were blocked with 10% donkey serum in PBS at room temperature for 30 min, followed by incubation with rabbit antiserum to phospho-YB-1 (Cell Signaling, 1:150) or p-ERK1/2 (Cell Signaling, 1:300), EGFR (Abcam, 1:100), and rat antiserum to Ki67 (eBioscience, 1:100, Karlsruhe, Germany). Nuclei were stained with YO-PRO-1 (Invitrogen, 1:2000). All washing steps were performed with PBS/Tween (PBST), and antibody incubations were performed in PBST with 0.05% BSA. The sections were analyzed with a confocal laser scanning microscope (Leica TCS SP; Leica Microsystems, Wetzlar, Germany) at 250× for P-YB-1 and 400× magnification for P-ERK1/2 as well as EGFR.

### 4.11. Statistics and Densitometry

Student’s *t*-test was used to compare the data between two groups. *p* < 0.05 was considered statistically significant (* *p* < 0.05; ** *p* < 0.01; *** *p* < 0.001). Densitometry analysis of the immunoblots was performed using LI-COR *Odyssey*^®^ *Fc* with Image Studio Lite software version 5.2. (Bad Homburg v. d. Hoeh, Germany) Mann-Whitney U analysis was performed for the in vivo study, and *p* < 0.0001 was considered significant at the 99% confidence level as shown by asterisks (****). We evaluated the correlation between the phosphorylation levels of ERK1/2 and YB-1 as well as the expression of EGFR using a Pearson correlation test.

## 5. Conclusions

The data presented in this study demonstrate the underlying signaling pathways involved in YB-1 phosphorylation in TNBC cells. The results provide a better understanding for investigating therapeutic approaches for other tumor entities with higher rates of *KRAS* mutation, e.g., pancreatic cancer and colorectal cancer, as well as in tumor entities with the hyperactivation of the PI3K/Akt pathway, which is of particular importance. The presented mechanistic study provides a rationale for dual targeting of MEK and PI3K as an efficient approach to block YB-1 phosphorylation at S102 and hamper its functions in cancer cells, such as resisting cell death and stimulating cell proliferation. To the best of our knowledge, this is the first study that shows that the MAPK/ERK and PI3K pathways regulate cell proliferation of TNBC cells through YB-1. Therefore, YB-1 might be an appropriate target to treat TNBC.

## Supplementary Materials

The following are available online at https://www.mdpi.com/2072-6694/12/10/2795/s1, Figure S1: Enhanced phosphorylation of YB-1 and ERK1/2 and expression of EGFR in breast cancer patient tissues, Figure S2: Genetic modification of YB-1 expression in MDA-MB-231 cells, Figure S3: The PI3K inhibitor LY294002 inhibits phosphorylation of YB-1 in *PTEN*-mutated prostate cancer cell line PC3, Figure S4: Phosphorylation of YB-1 is independent of Akt isoforms in *KRAS(G13D)*-mutated MDA-MB-231 cells, Figure S5: KRAS(G13D) knockdown leads to the MAPK/ERK-dependent inhibition of YB-1 phosphorylation and the PI3K-dependent activation of Akt, Figure S6: Uncropped western blot figures.
