# Supplementary material for: Blocking Y-Box Binding Protein-1 through Simultaneous Targeting of PI3K and MAPK in Triple Negative Breast Cancers
Source: Cancers (Basel). 2020 Sep 29;12(10):2795. doi: 10.3390/cancers12102795 (PMC7601769; doi:10.3390/cancers12102795)
Supplement: Supplementary file 1 [file cancers-12-02795-s001.pdf]

Blocking Y-box Binding Protein-1 Through Simultaneous Targeting of PI3K and MAPK in Triple Negative Breast Cancers

Aadhya Tiwari, Mari Iida, Corinna Kosnopfel, Mahyar Abbariki, Apostolos Menegakis, Birgit Fehrenbacher, Julia Maier, Martin Schaller, Sara Y. Brucker, Deric L. Wheeler, Paul M. Harari, Ulrich Rothbauer, Birgit Schittek, Daniel Zips, Mahmoud Toulany

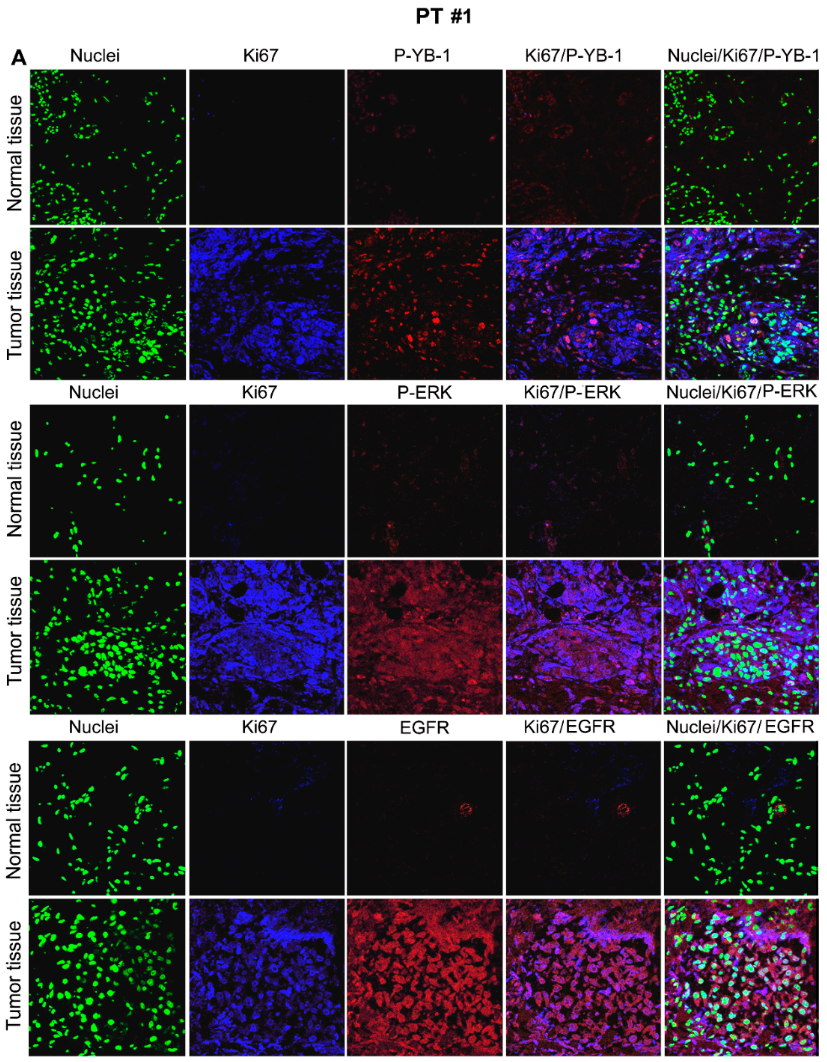

B

Pts. ID	Cancer type	Grade	Stage	Metastasis	Lymphatic invasion	Vascular invasion	Estrogen receptor	Progestron receptor	Her2 receptor
PT1	Duct. inv.	2	pT2	Yes	L1	no	negative	negative	positive
PT2	Duct. inv.	3	pT2	Yes	L1	V1	negative	negative	positive
PT3	Duct. inv.	3	pT4b	Yes	L1	no	positive	positive	negative
PT4	-	-	-	-	-	-	-	-	-
PT5	Duct. inv.	3	pT3	Yes	L1	no	negative	negative	negative
PT6	Duct. inv.	2	pT2	Yes	L1	no	negative	negative	negative

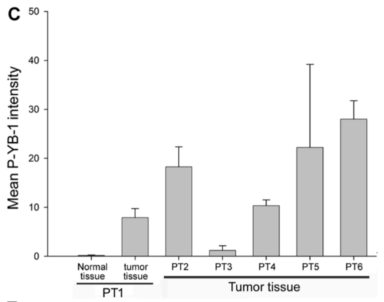

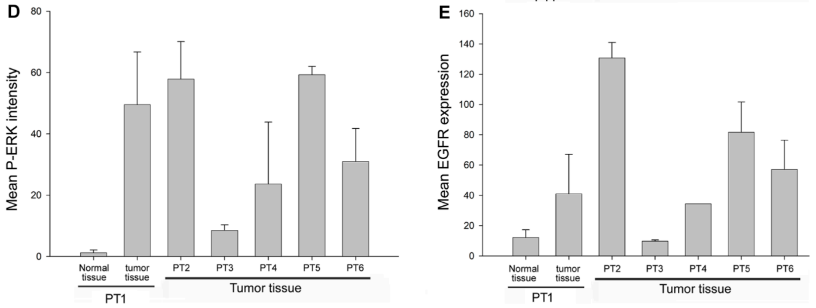

Figure S1. Enhanced phosphorylation of YB-1 and ERK1/2 and expression of EGFR in breast cancer patient tissues. (**A**) Paraffin-embedded sections were used for immunofluorescence staining of P-ERK1/2 (T202/Y204), P-YB-1 (S102) and EGFR in tumor tissues and the normal tissue from patient number 1 (PT1). Nuclei were stained with YO-PRO. Ki67 was stained as an indicator of cell proliferation. The sections were analyzed with a confocal laser scanning microscope at 250× magnification for P-YB-1 and 400× magnification for P-ERK1/2 as well as EGFR. **(B)** Molecular pathology of tumor tissues from 6 breast cancer patients. The mean intensity of fluorescence staining for P-YB-1 **(C)**, P-ERK1/2 **(D)** and EGFR **(E)** was analyzed in 3 randomly selected areas in each image. Pearson correlation coefficient tests revealed a positive moderate correlation between P-ERK1/2 and P-YB-1 (r = 0.51), a positive moderate correlation between P-YB-1 and EGFR (r = 0.61) and a positive strong correlation between P-ERK1/2 and EGFR (r = 0.81). **(F)** Paraffin-embedded sections were used for comparing the staining of P-YB-1 and YB-1 in tumor tissues and the normal tissue from patient number 1 (PT1). Nuclei were stained with YO-PRO. Ki67 was stained as an indicator of cell proliferation. The sections were analyzed with a confocal laser scanning microscope at 400x magnification.

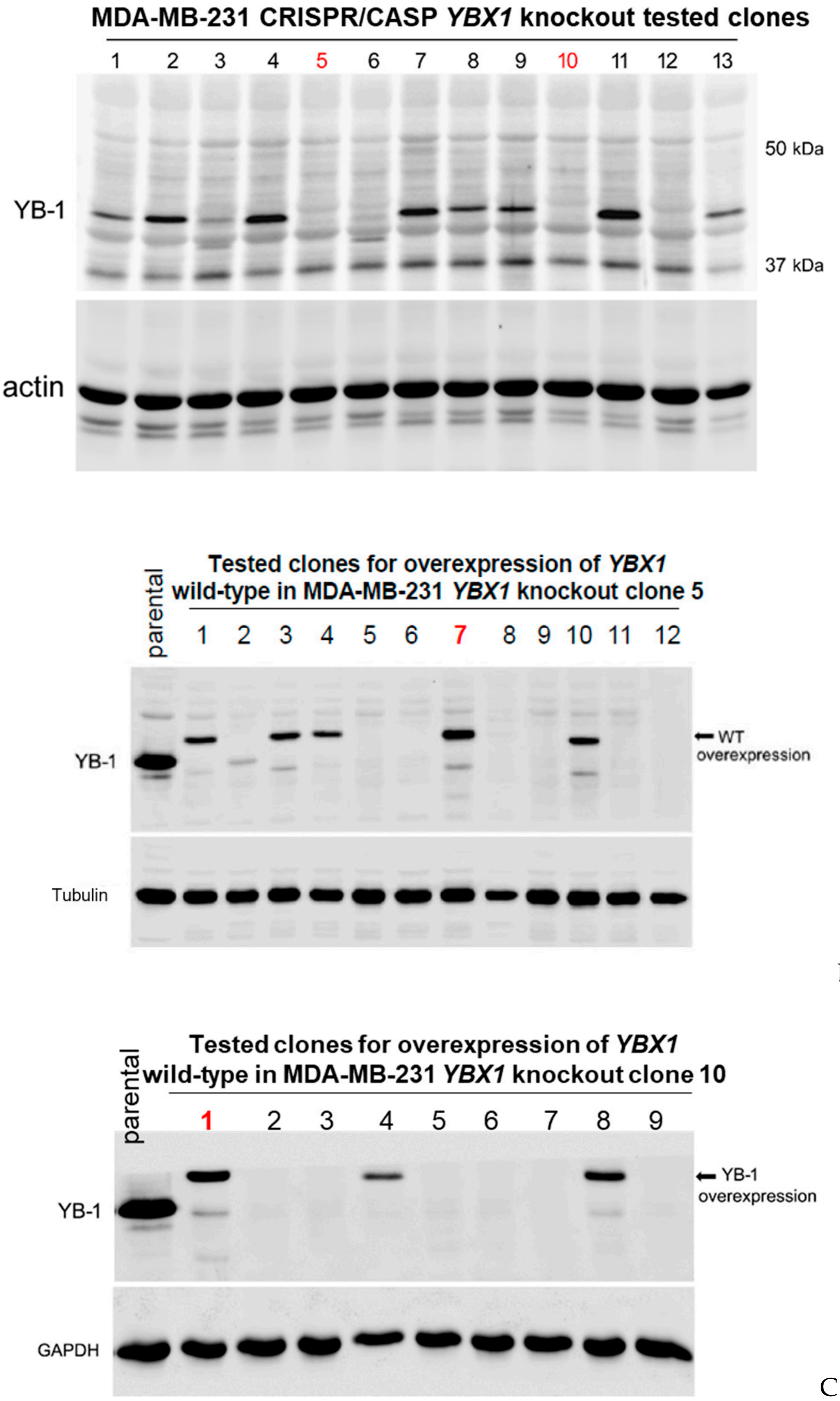

Figure S2. Genetic modification of YB-1 expression in MDA-MB-231 cells. (**A**) YB-1 knockout was performed using CRISPR/Cas9. Knockout efficiency was tested by Western blotting in indicated clones. Wild-type human YB-1 was reconstructed in MDA-MB-231 YBX1 knockout clone 5. (**B**) as well as in MDA-MB-231 YBX1 knockout clone 10 (**C**) and the expression level was tested by Western blotting in indicated clones.

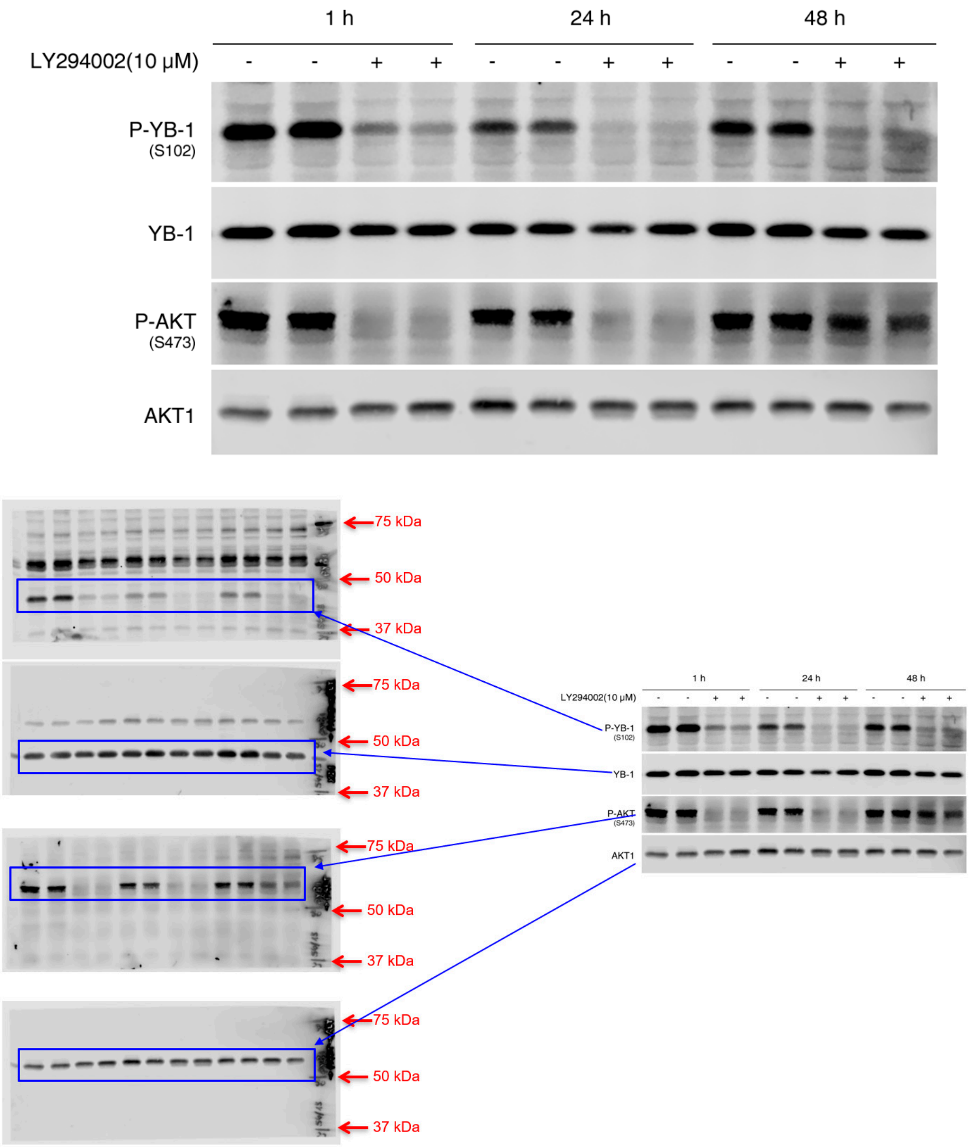

Figure S3. The PI3K inhibitor LY294002 inhibits phosphorylation of YB-1 in *PTEN* mutated prostate cancer cell line PC3. Cells were treated with LY294002 for indicated time-points. Thereafter, protein samples were isolated and subjected to SDS-PAGE. The phosphorylation and expression of the indicated proteins were analyzed by western blotting. Protein samples loaded from cells with the same treatment conditions were isolated from 2 parallel cultures.

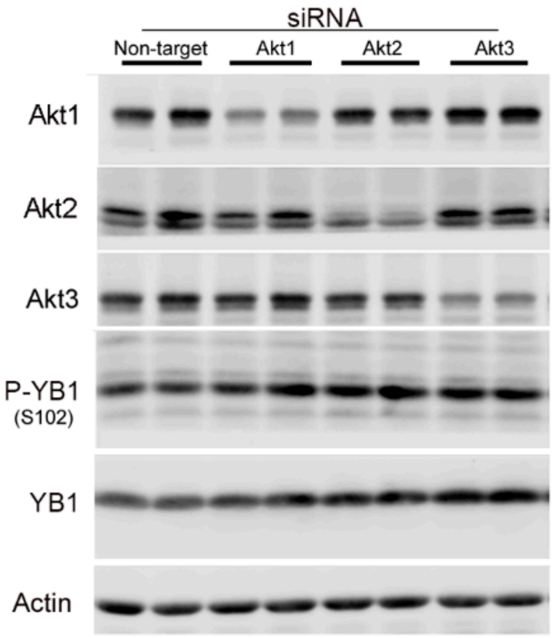
siRNA
Non-target
Akt1
Akt2
Akt3
Akt1
Akt2
Akt3
P-YB1
(S102)
YB1
Actin

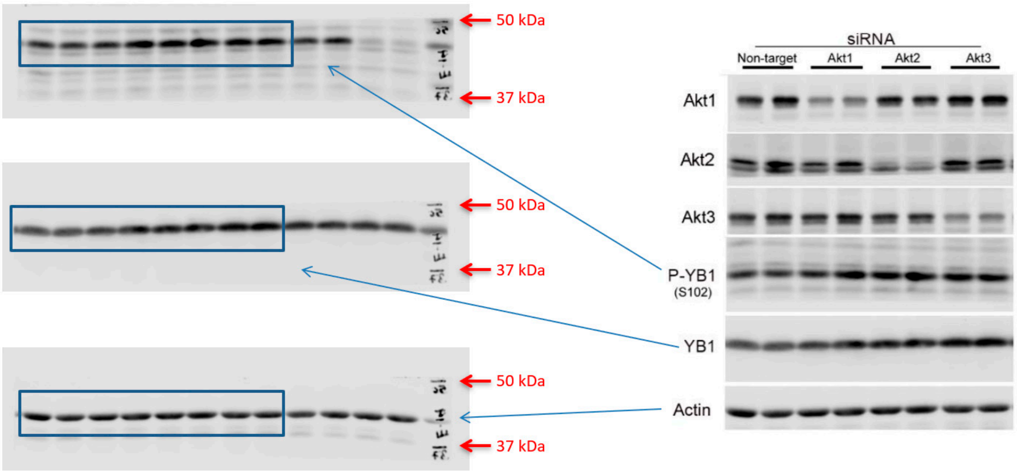
50 kDa
37 kDa
50 kDa
37 kDa
50 kDa
37 kDa
siRNA
Non-target
Akt1
Akt2
Akt3
Akt1
Akt2
Akt3
P-YB1
(S102)
YB1
Actin

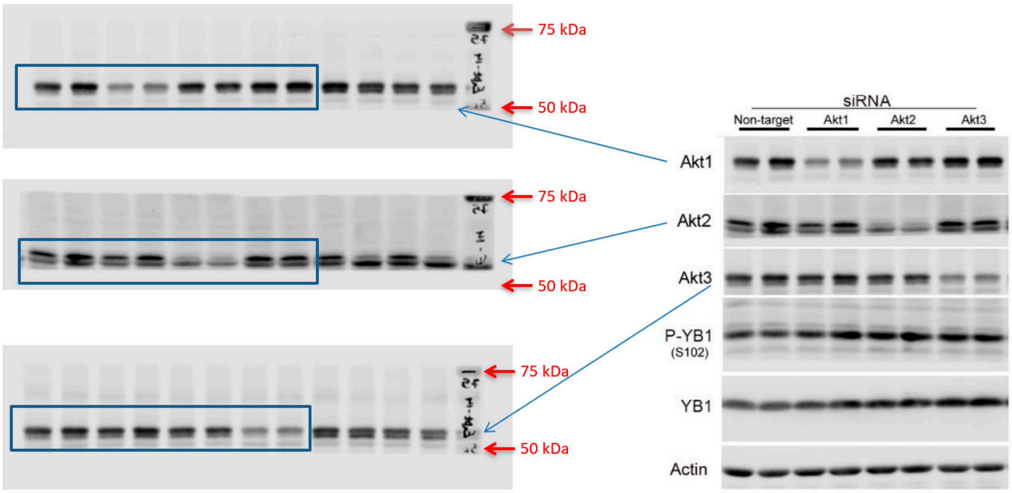
75 kDa
50 kDa
75 kDa
50 kDa
75 kDa
50 kDa
siRNA
Non-target
Akt1
Akt2
Akt3
Akt1
Akt2
Akt3
P-YB1
(S102)
YB1
Actin

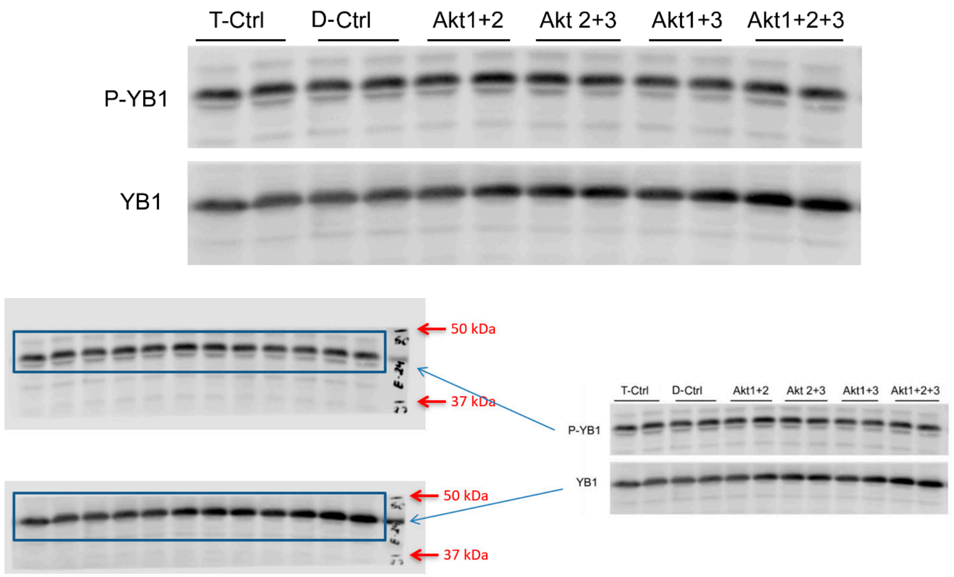

Figure S4. Phosphorylation of YB-1 is independent of AKT isoforms in *KRAS(G13D)* mutated MDA-MB-231 cells. Cells were transfected with indicated siRNA**.** Seventy-two hours after transfection, protein samples were isolated. The phosphorylation and expression of YB-1 as well as the expression of AKT isoforms were analyzed by Western blotting. In panel A, actin was detected as loading control. Protein samples exposed to the same treatment conditions were isolated from 2 parallel cultures. D-Ctrl: control siRNA for dual knockdown, T-Ctrl: control siRNA for triple knockdown.

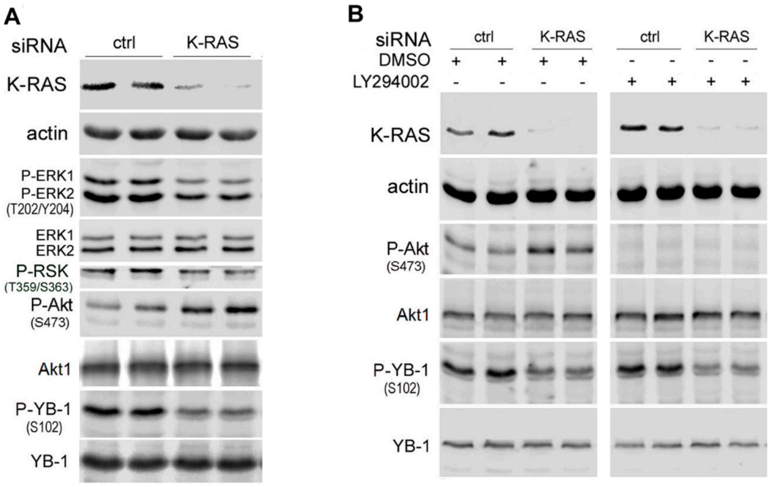

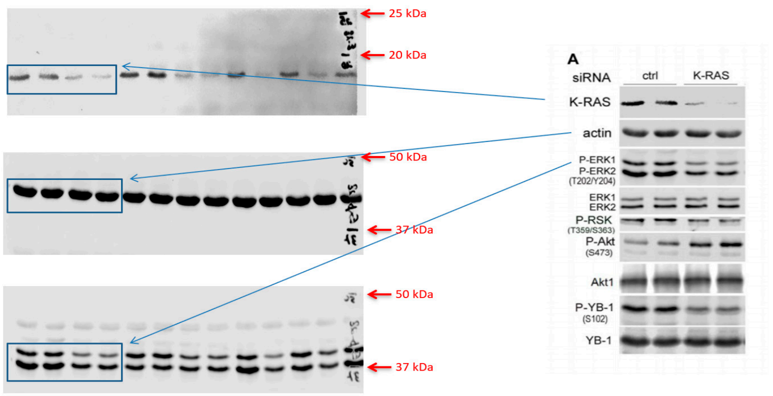
25 kDa
20 kDa
50 kDa
37 kDa
50 kDa
37 kDa
A
siRNA
ctrl
K-RAS
K-RAS
actin
P-ERK1
P-ERK2
(T202/Y204)
ERK1
ERK2
P-RSK
(T359/S363)
P-Akt
(S473)
Akt1
P-YB-1
(S102)
YB-1

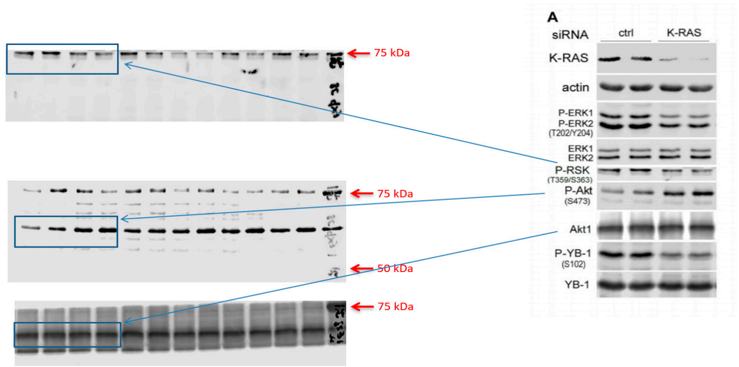
75 kDa
75 kDa
50 kDa
75 kDa
A
siRNA
ctrl
K-RAS
K-RAS
actin
P-ERK1
P-ERK2
(T202/Y204)
ERK1
ERK2
P-RSK
(T359/S363)
P-Akt
(S473)
Akt1
P-YB-1
(S102)
YB-1

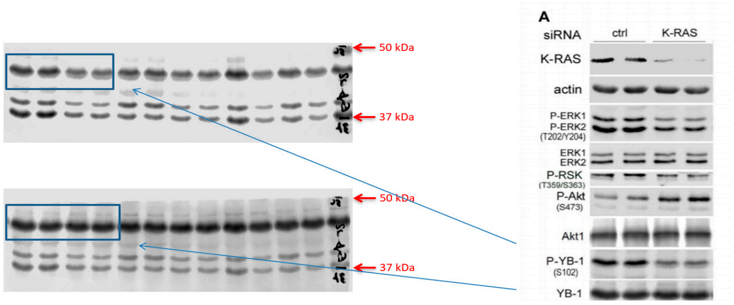
50 kDa
37 kDa
50 kDa
37 kDa
A
siRNA
ctrl
K-RAS
K-RAS
actin
P-ERK1
P-ERK2
(T202/Y204)
ERK1
ERK2
P-RSK
(T359/S363)
P-Akt
(S473)
Akt1
P-YB-1
(S102)
YB-1

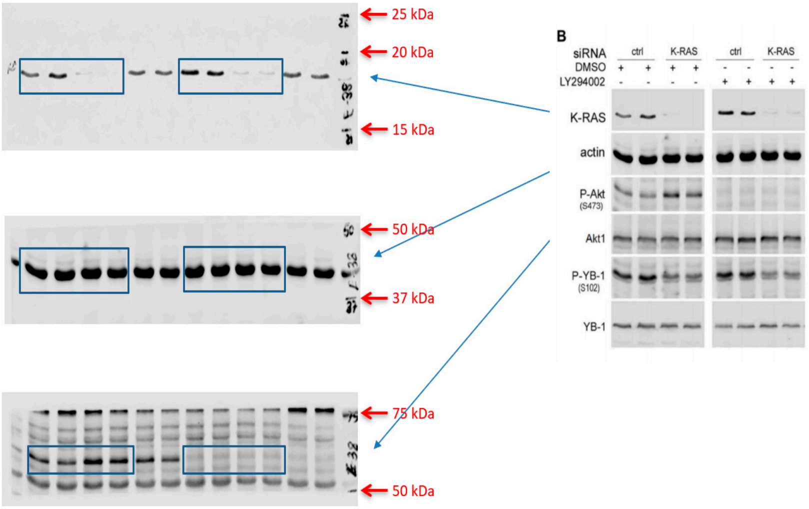

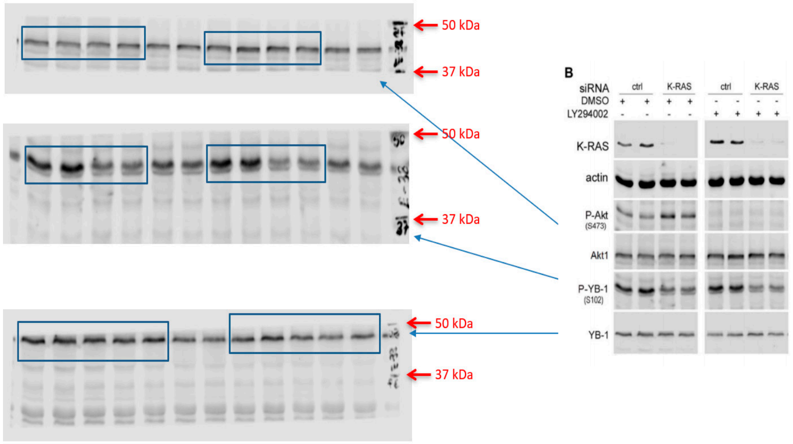

Figure S5. KRAS(G13D) knockdown leads to the MAPK/ERK-dependent inhibition of YB-1 phosphorylation and the PI3K-dependent activation of Akt. MDA-MB-231 cells were transfected with control-siRNA (ctrl-siRNA) and KRAS-siRNA (**A**) or transfected with indicated siRNA and treated with PI3K inhibitor LY284002 (10 µM) (**B**). Seventy-two hours after transfection and 2 h after treatment with the indicated inhibitors, protein samples were isolated. The phosphorylation and expression of the indicated proteins were analyzed by western blotting. Protein samples exposed to the same treatment conditions were isolated from 2 parallel cultures.

Fig. 1

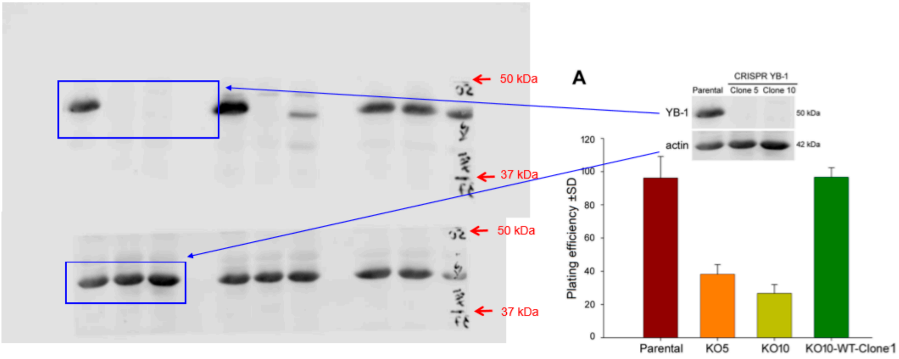

Fig. 2A

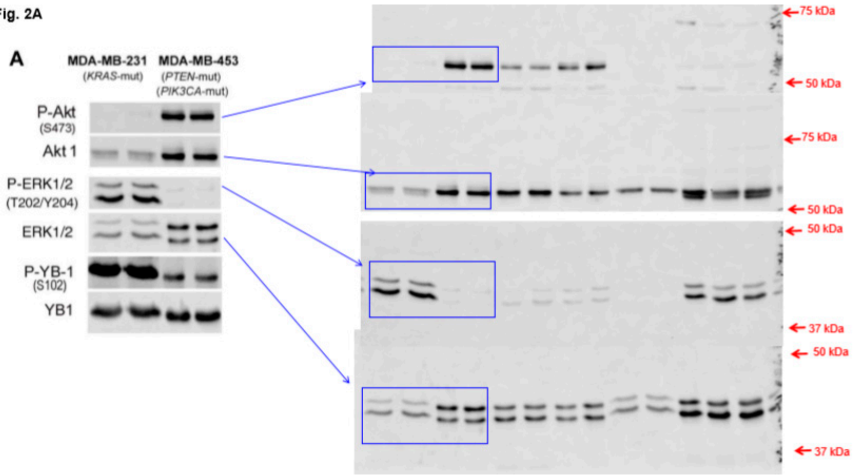

Fig. 2A

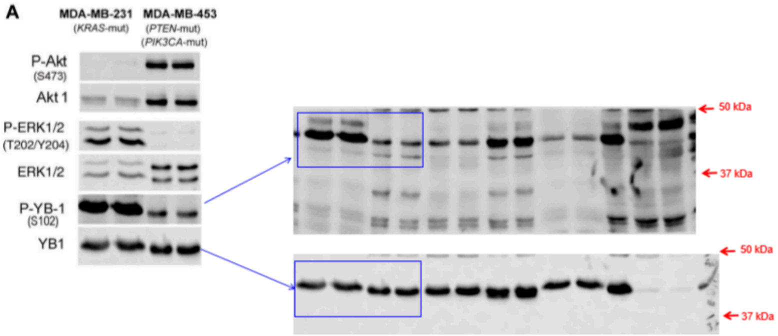

Fig. 2B

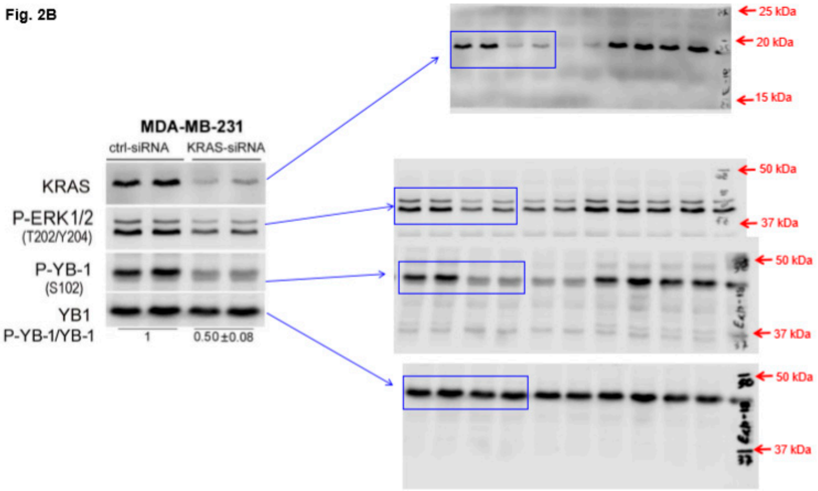

Fig. 2B

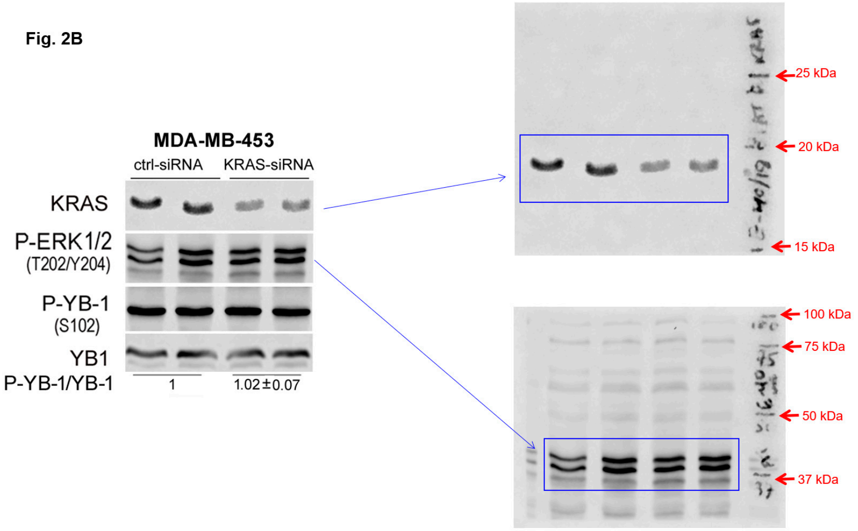

Fig. 2B

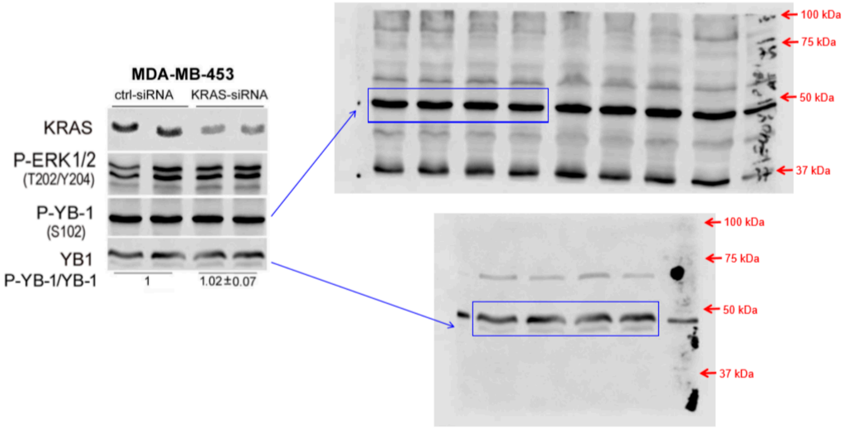

Fig. 2C

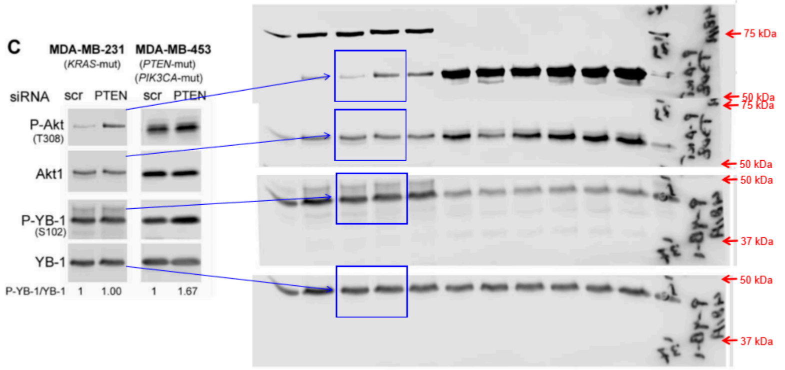

Fig. 2C

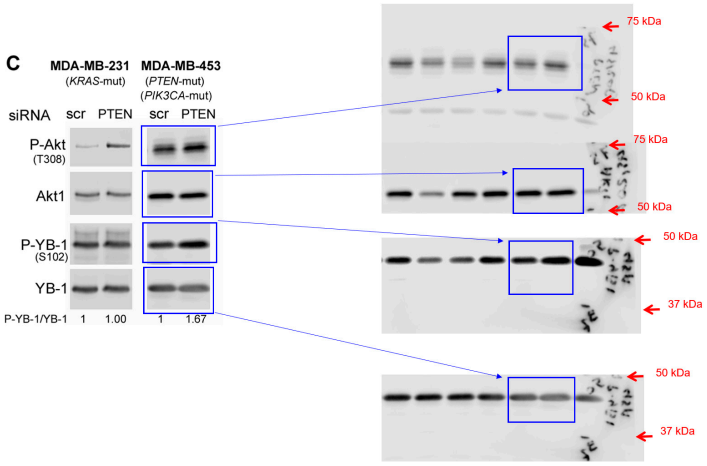

Fig. 2D

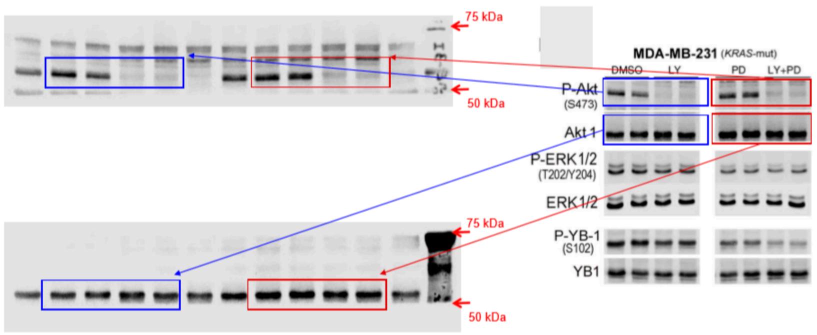

Fig. 2D

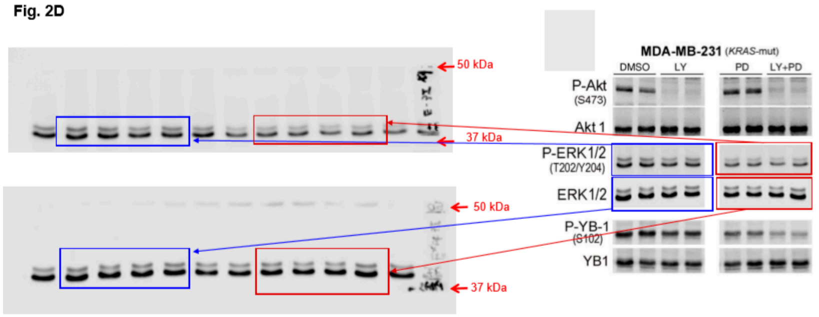

Fig. 2D

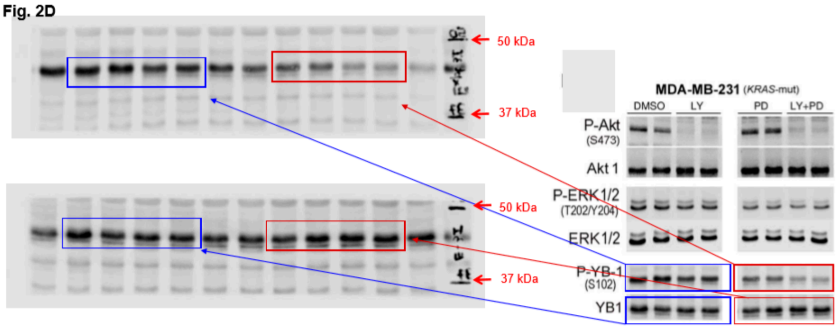

Fig. 2D

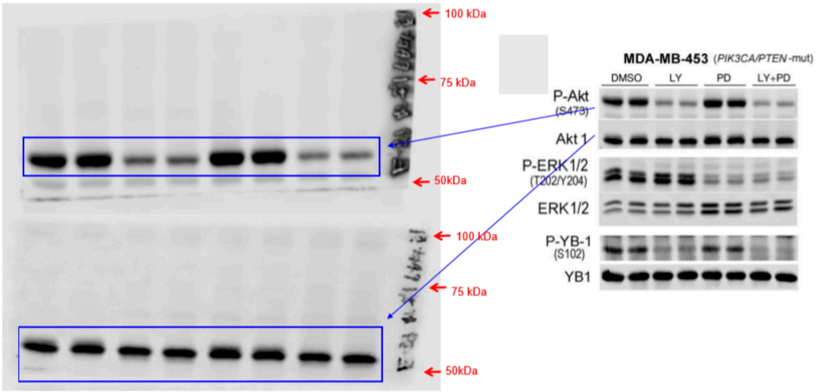

Fig. 2D

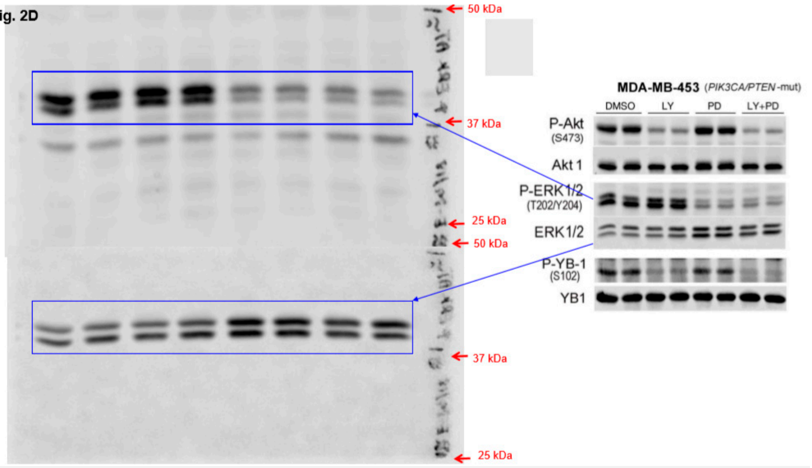

Fig. 2D

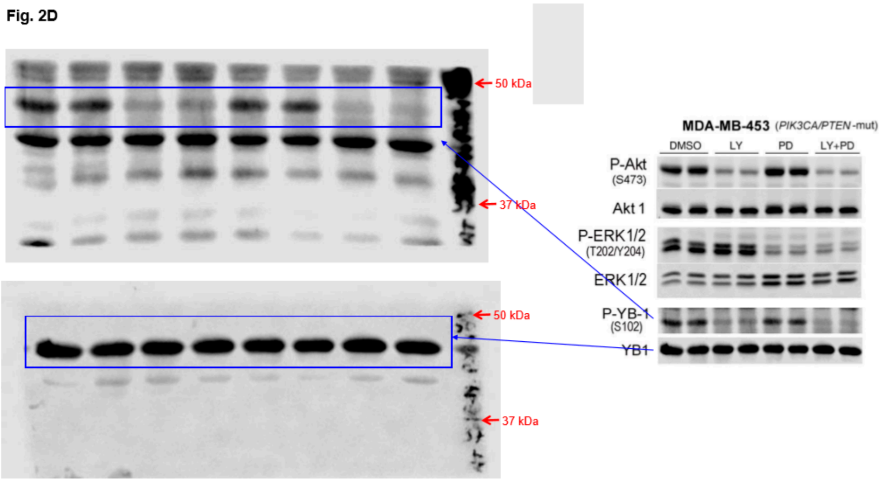

Fig. 3A

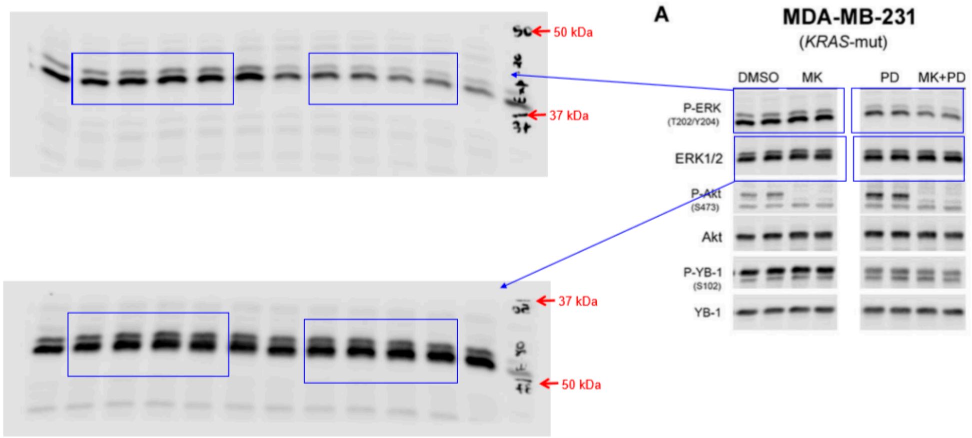

Fig. 3A

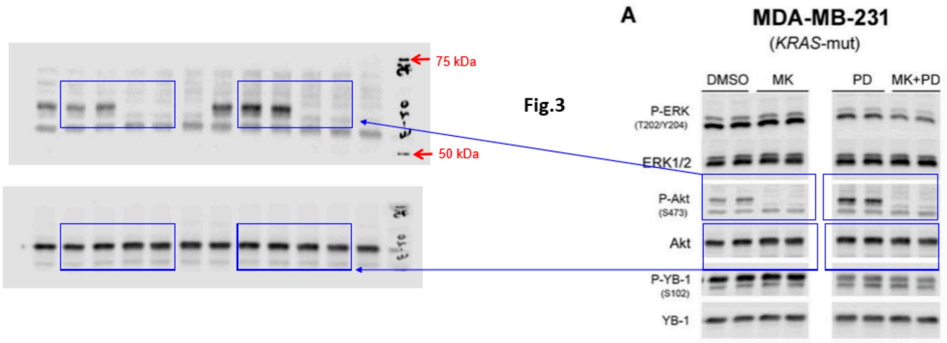

Fig. 3A

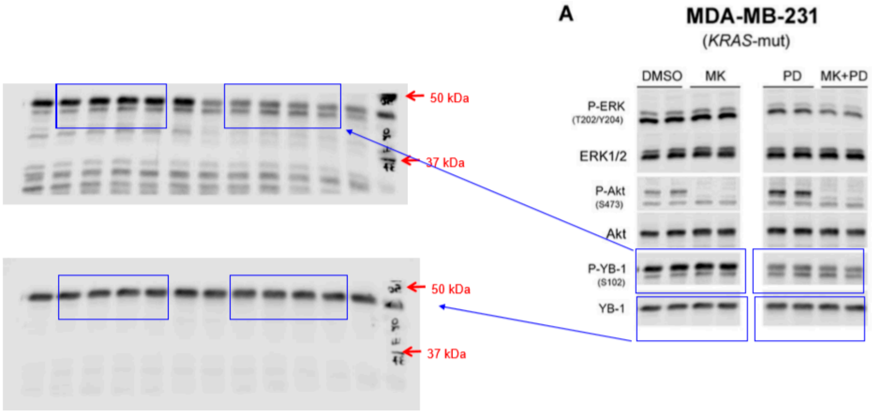

Fig. 3A

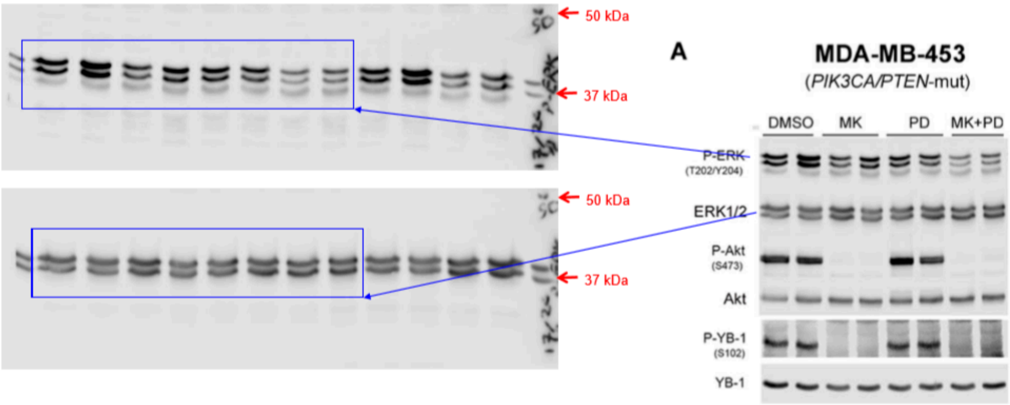

Fig. 3A

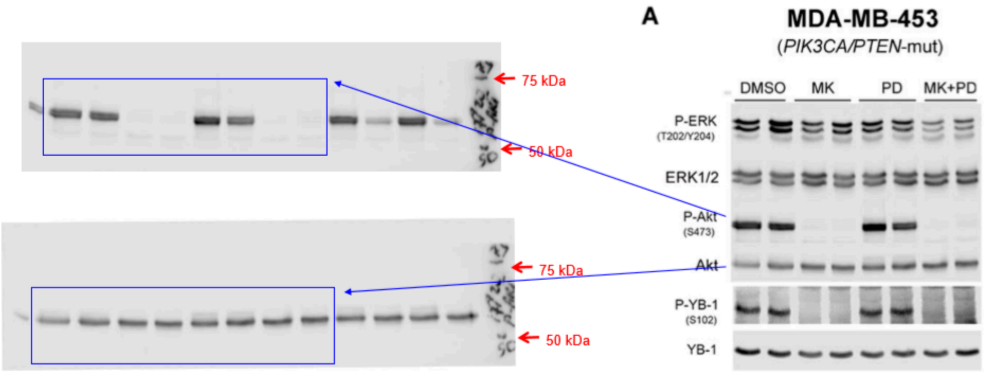

Fig. 3A

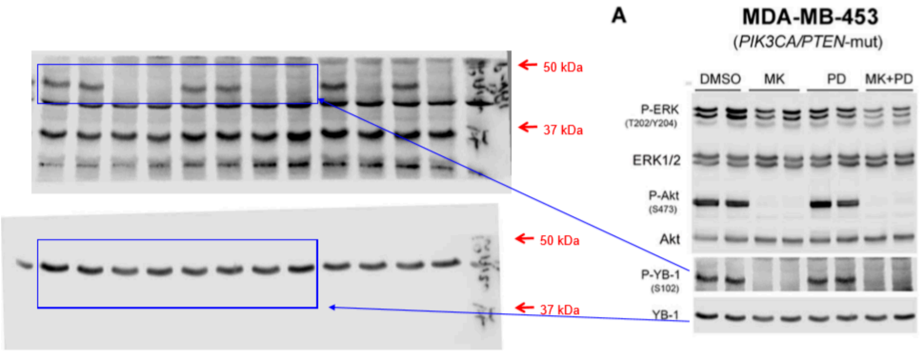

Fig. 3C

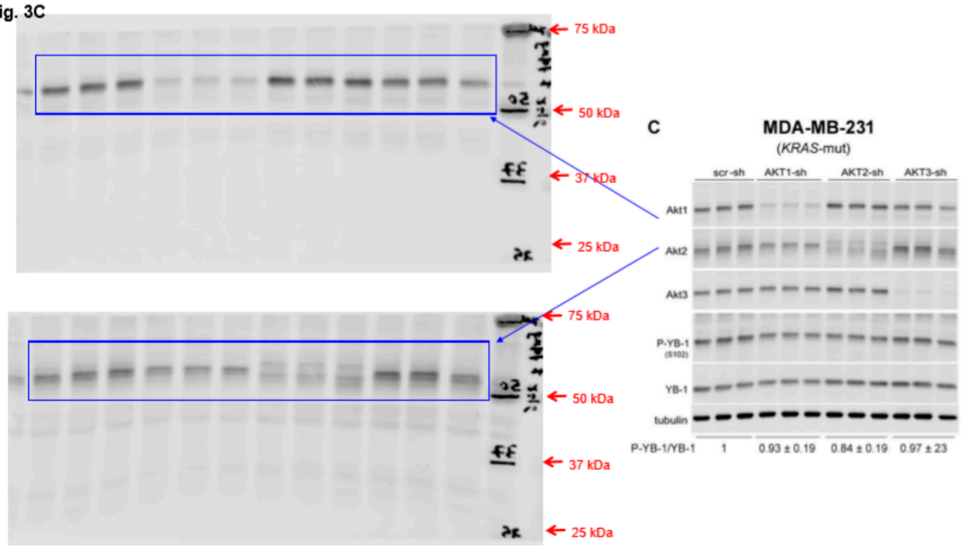

Fig. 3C

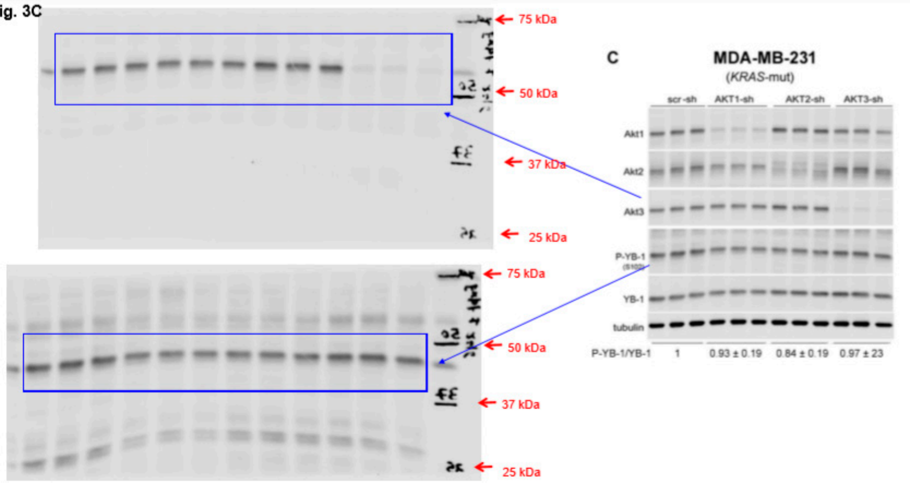

Fig. 3C

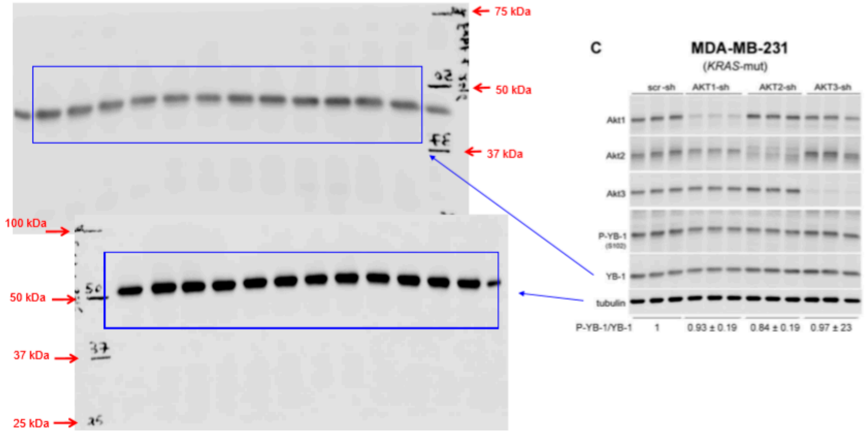

Fig. 3D

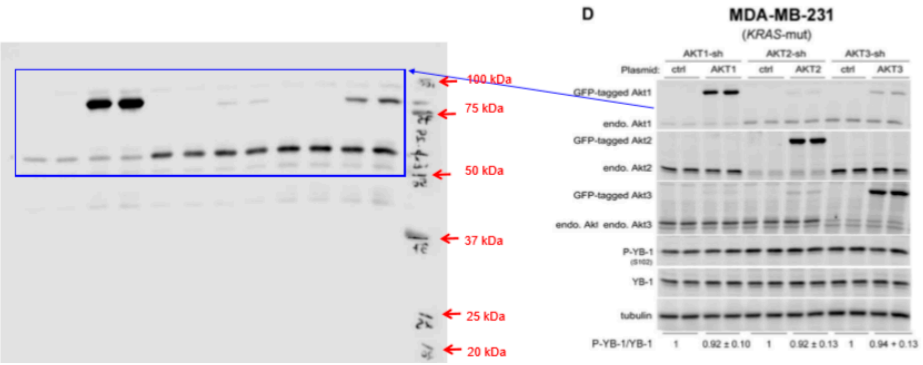

Fig. 3D

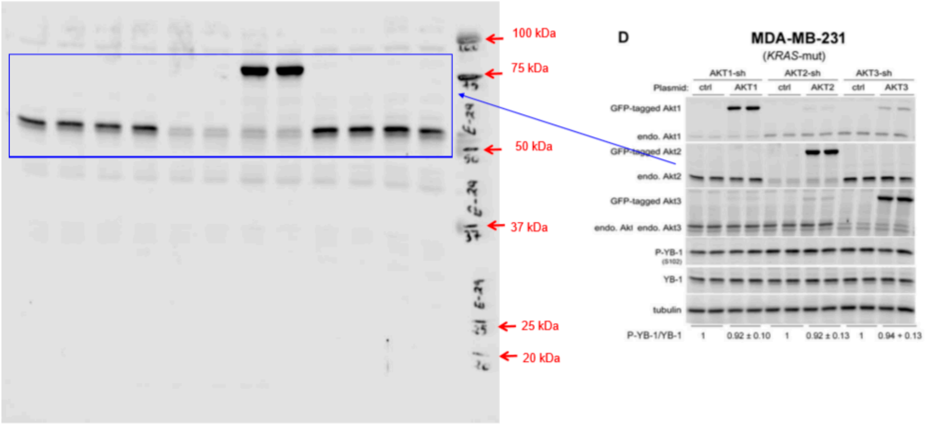

Fig. 3D

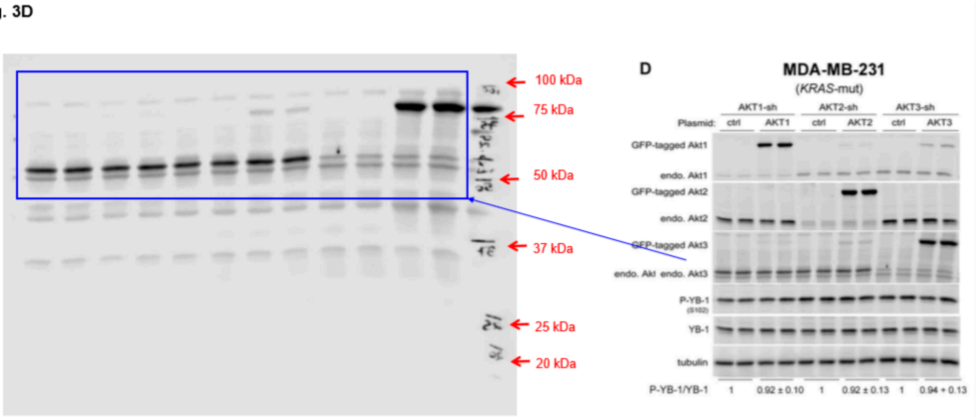

Fig. 3D

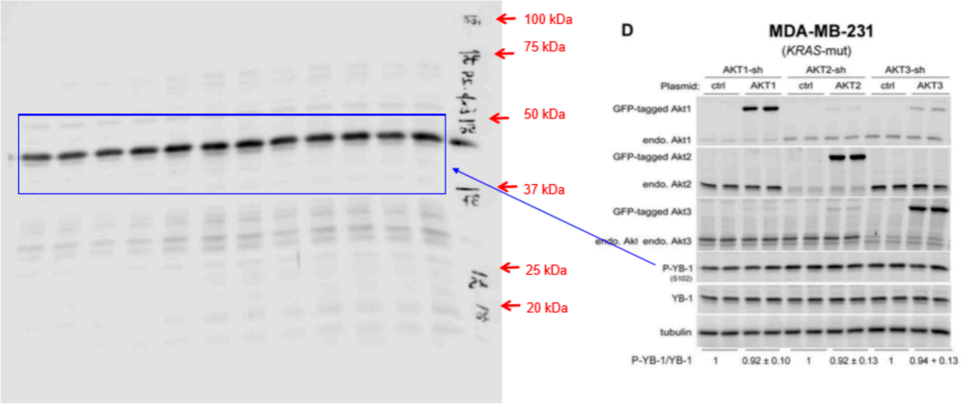

Fig. 3D

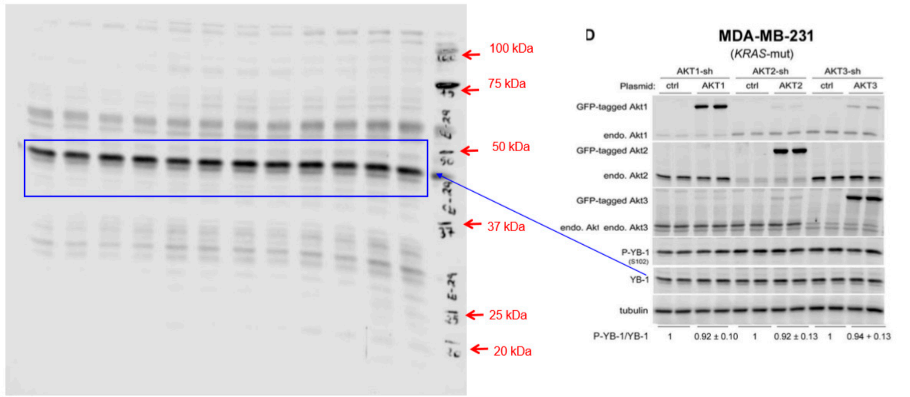

Fig. 3D

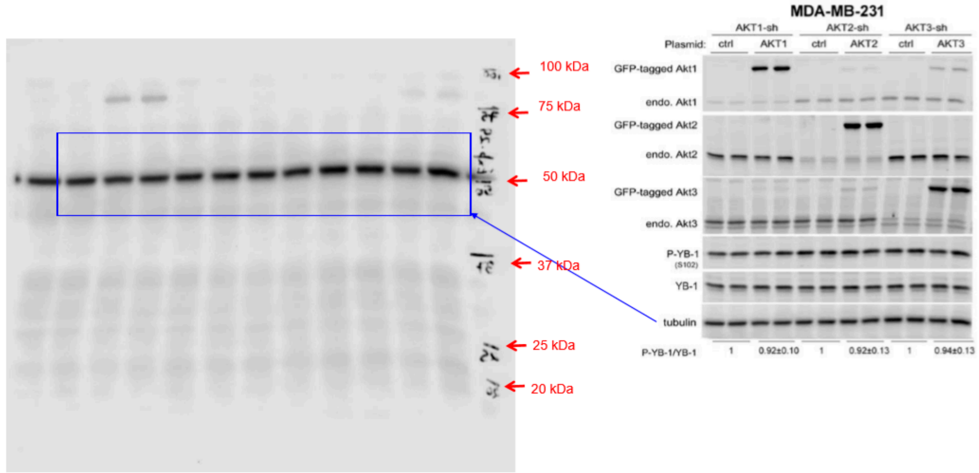

Fig. 3E

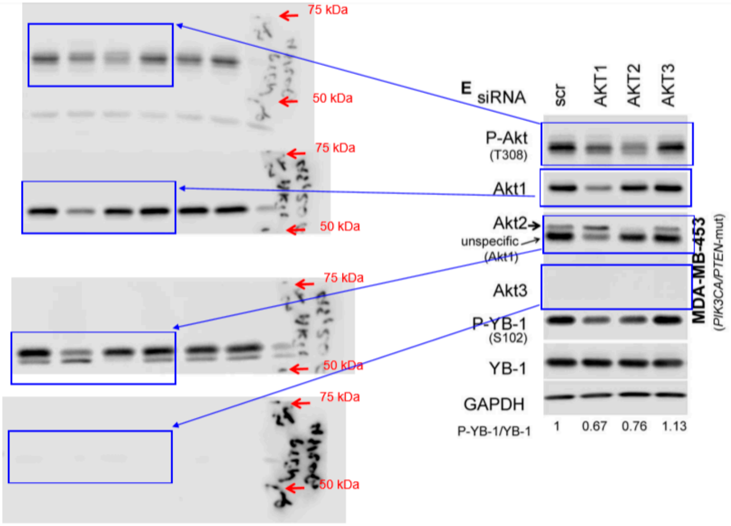

Fig. 3E

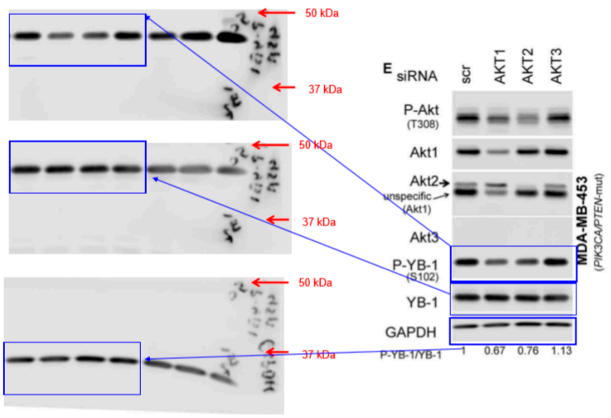

Fig. 4C

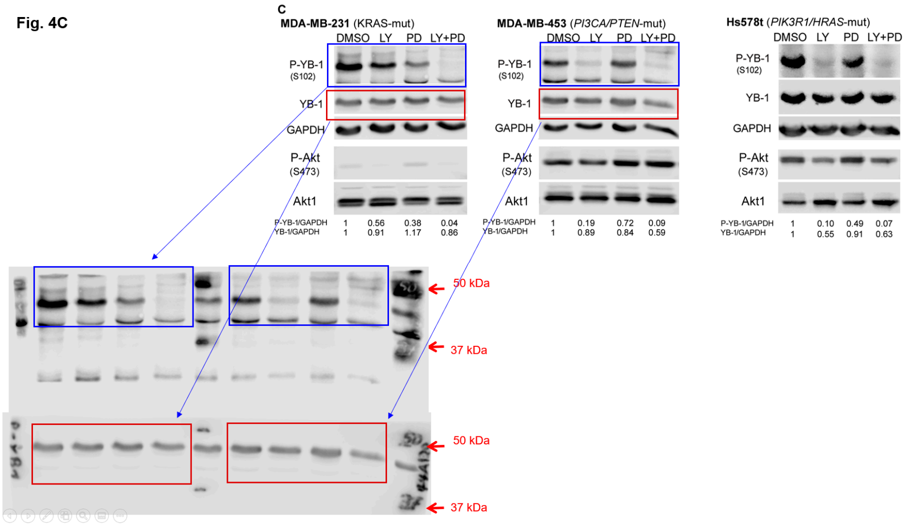

Fig. 4C

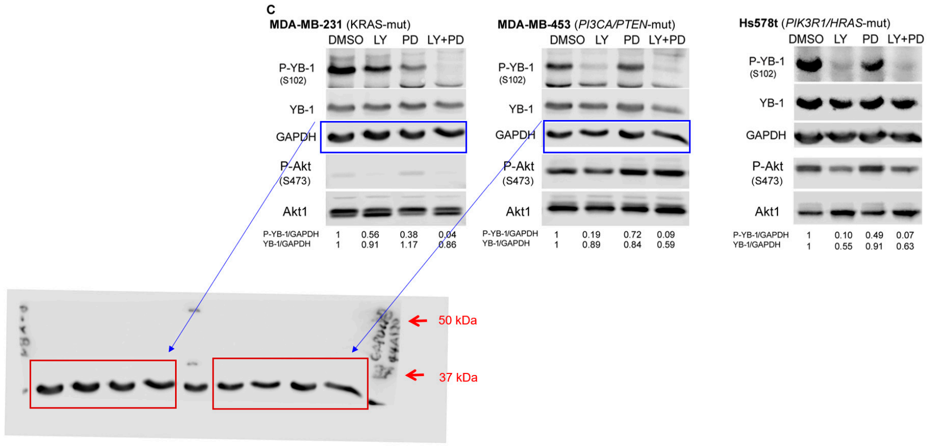

Fig. 4C

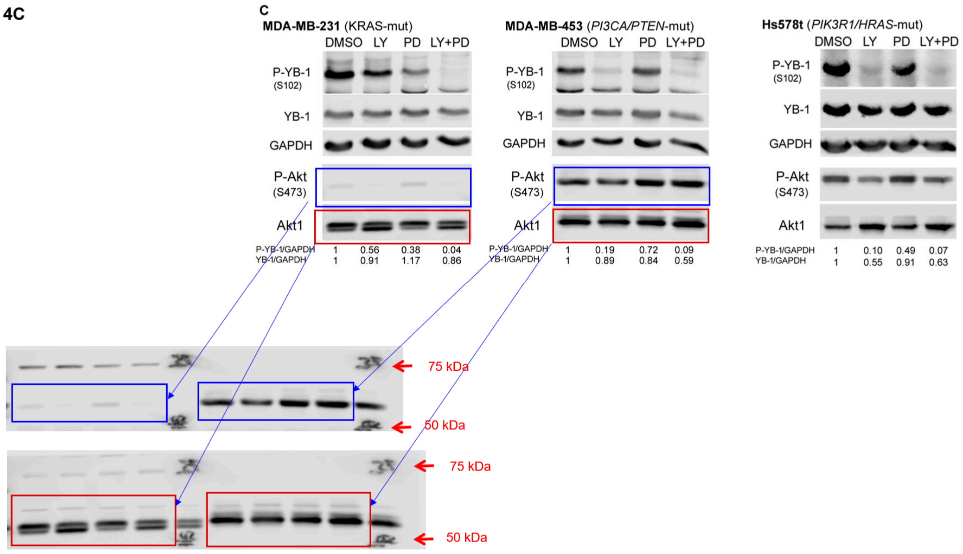

Fig. 4C

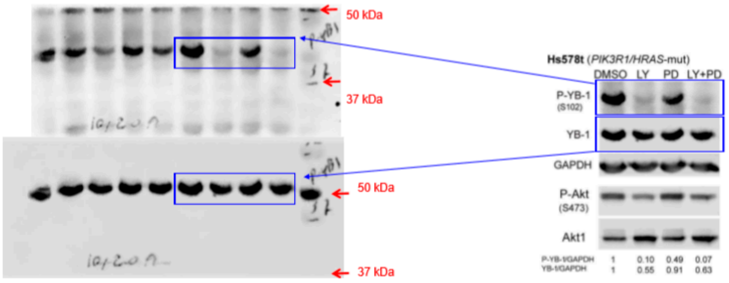

Fig. 4C

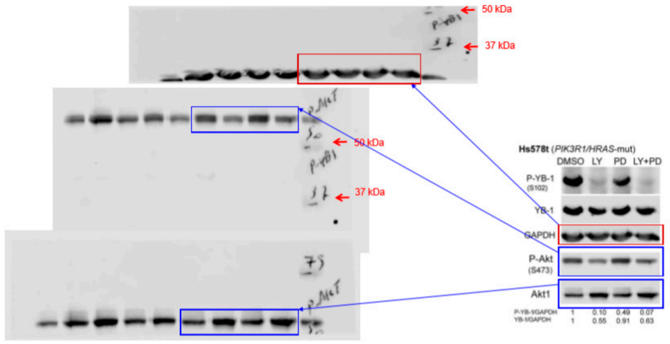

Fig. 4F

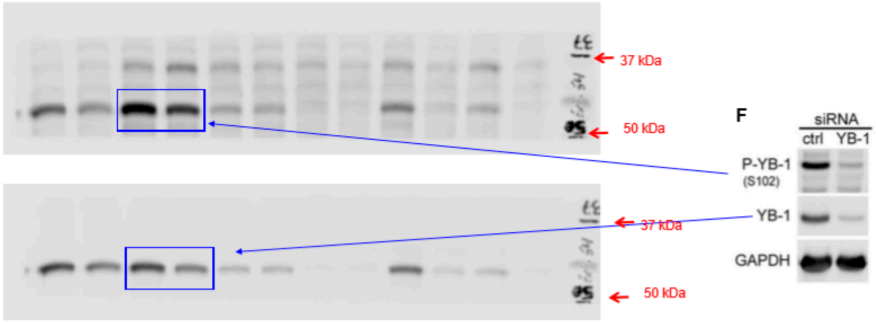

Fig. 4F

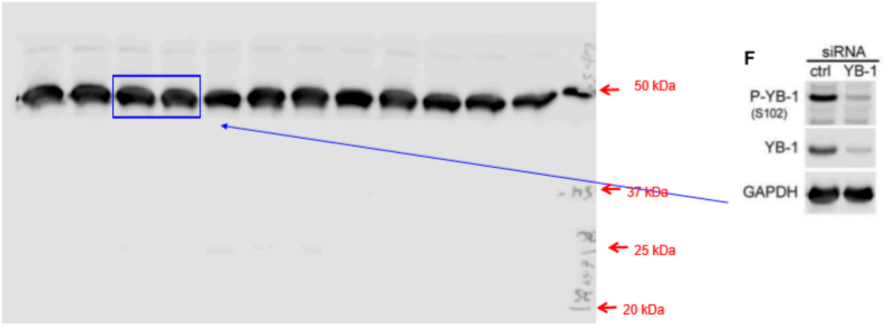

Figure S6. Uncropped Western blot figures.

© 2020 by the authors. Licensee MDPI, Basel, Switzerland. This article is an open access article distributed under the terms and conditions of the Creative Commons Attribution (CC BY) license (http://creativecommons.org/licenses/by/4.0/).
